# Supplementary figures and images for: The Calpain-7 protease functions together with the ESCRT-III protein IST1 within the midbody to regulate the timing and completion of abscission
Source: eLife. 2023 Sep 29;12:e84515. doi: 10.7554/eLife.84515 (PMC10586806; doi:10.7554/eLife.84515)

MW  
(kDa)

250

150

100

75

50

37

25

20

15

10

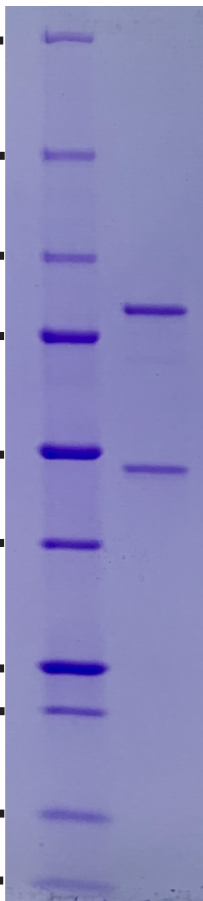

◀ CAPN7

◀ IST1

Supplement: Figure 3—source data 1. [file elife-84515-fig3-data1.zip › Figure 3-source data-1/Figure-3B-uncroppedgel.pdf]

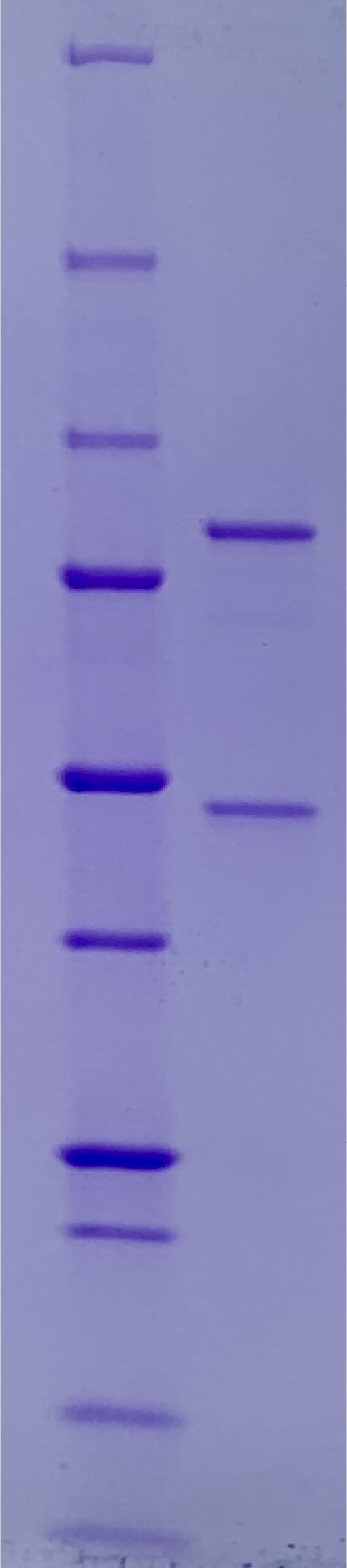

Supplement: Figure 3—source data 1. [file elife-84515-fig3-data1.zip › Figure 3-source data-1/Raw image/Figure 3B gel raw.png]

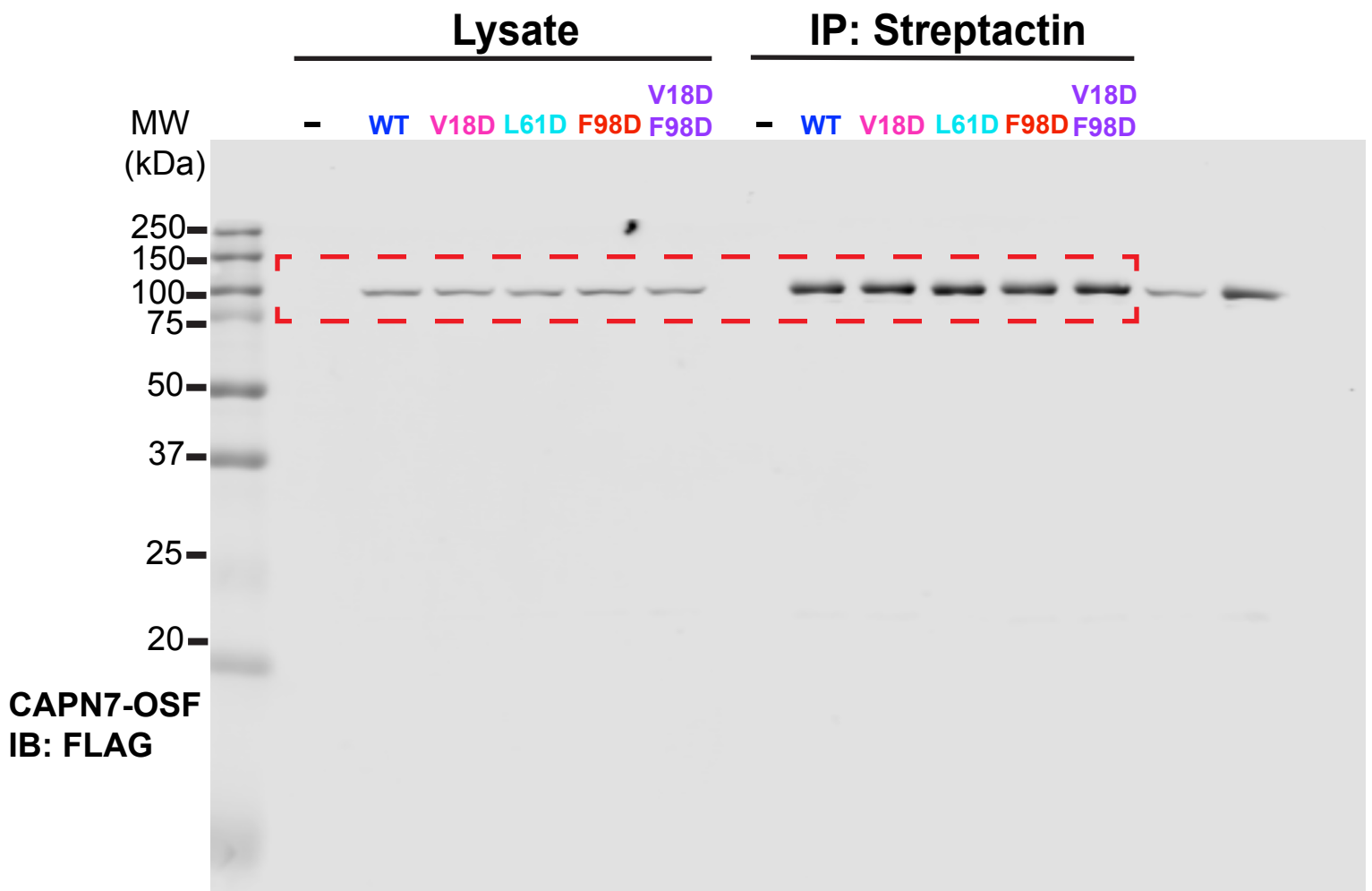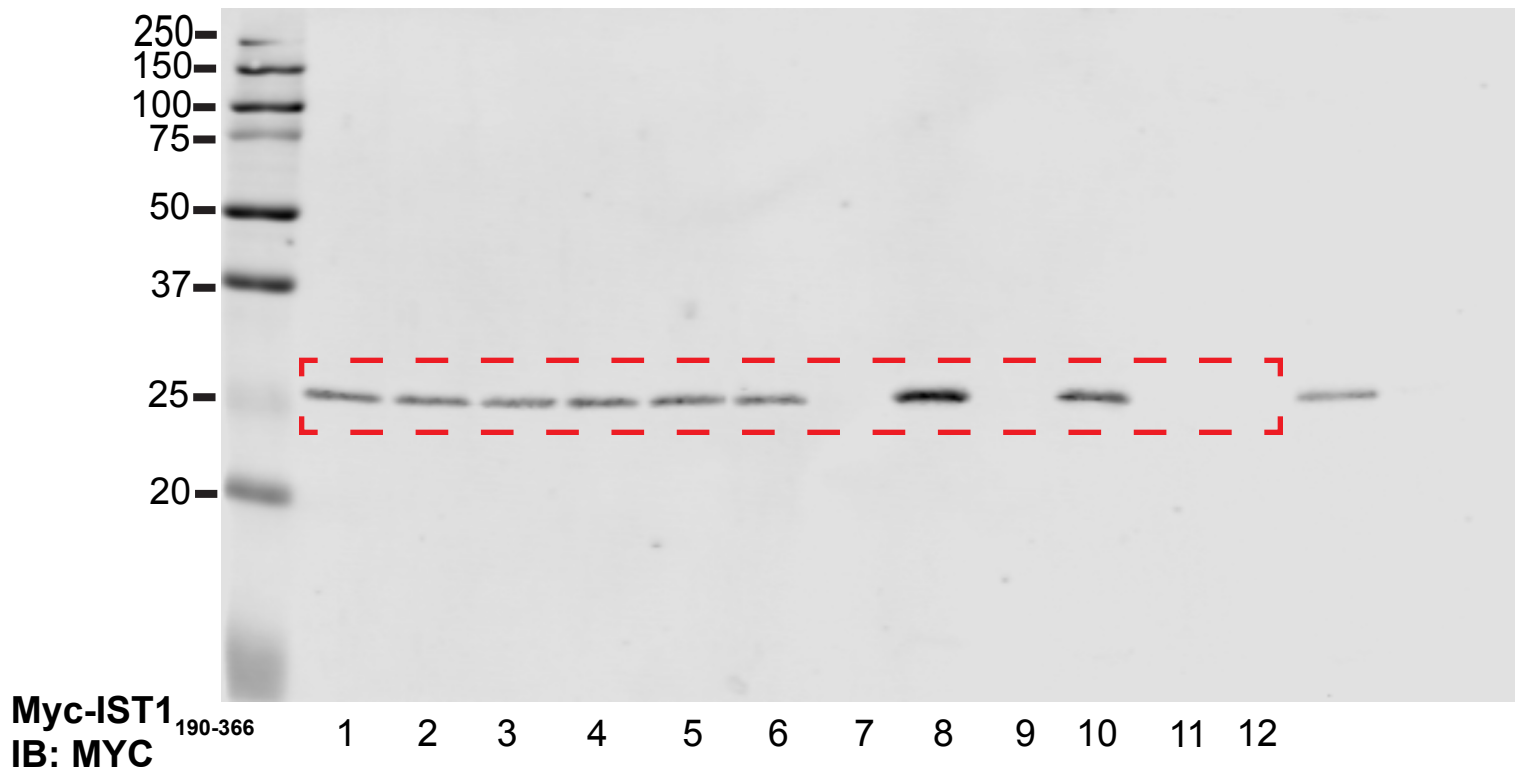

Supplement: Figure 3—source data 2. [file elife-84515-fig3-data2.zip › Figure 3-source data-2/Figure 3C-uncropped blots.pdf]

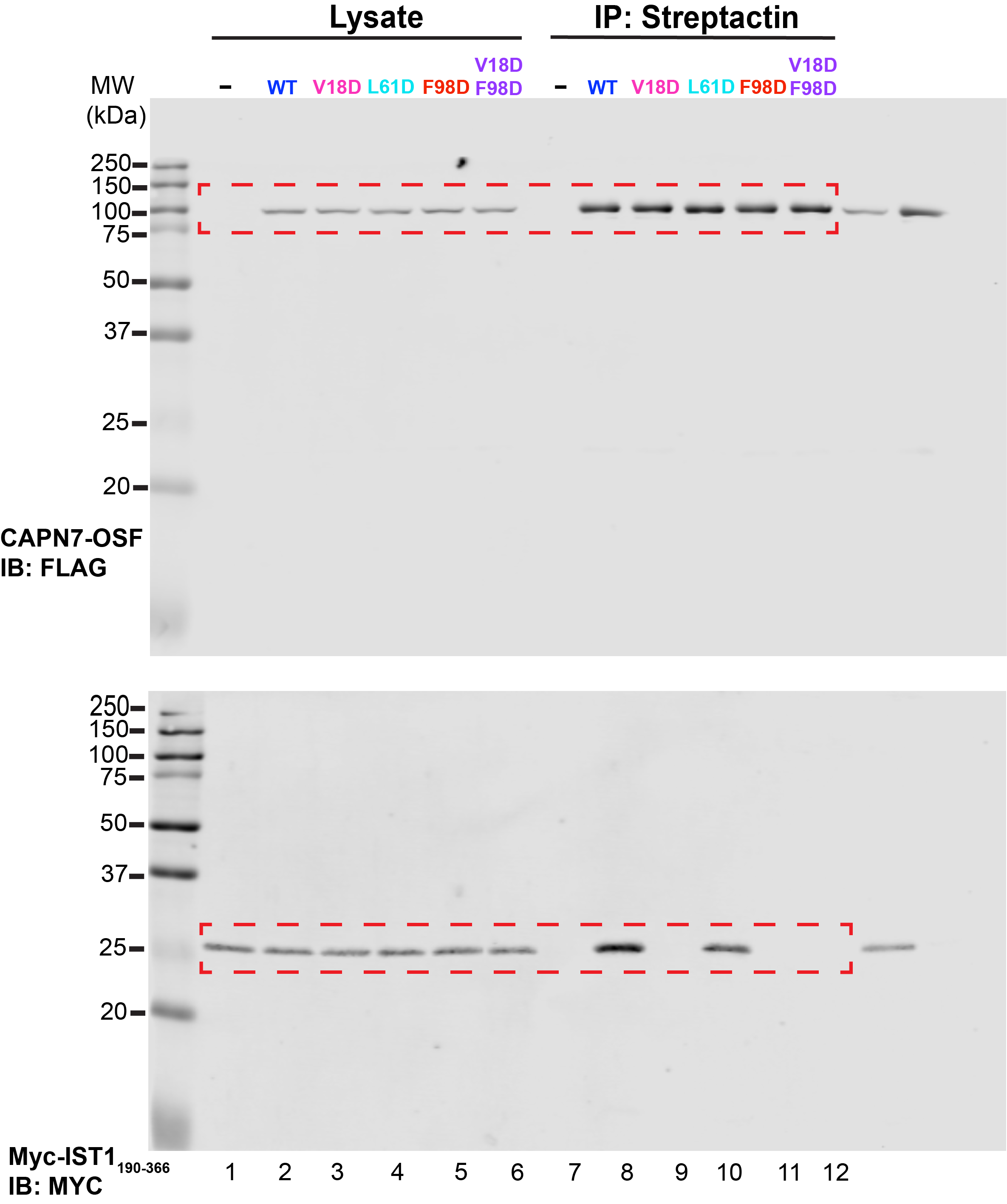

Supplement: Figure 3—source data 2. [file elife-84515-fig3-data2.zip › Figure 3-source data-2/Figure 3C-uncropped blots.png]

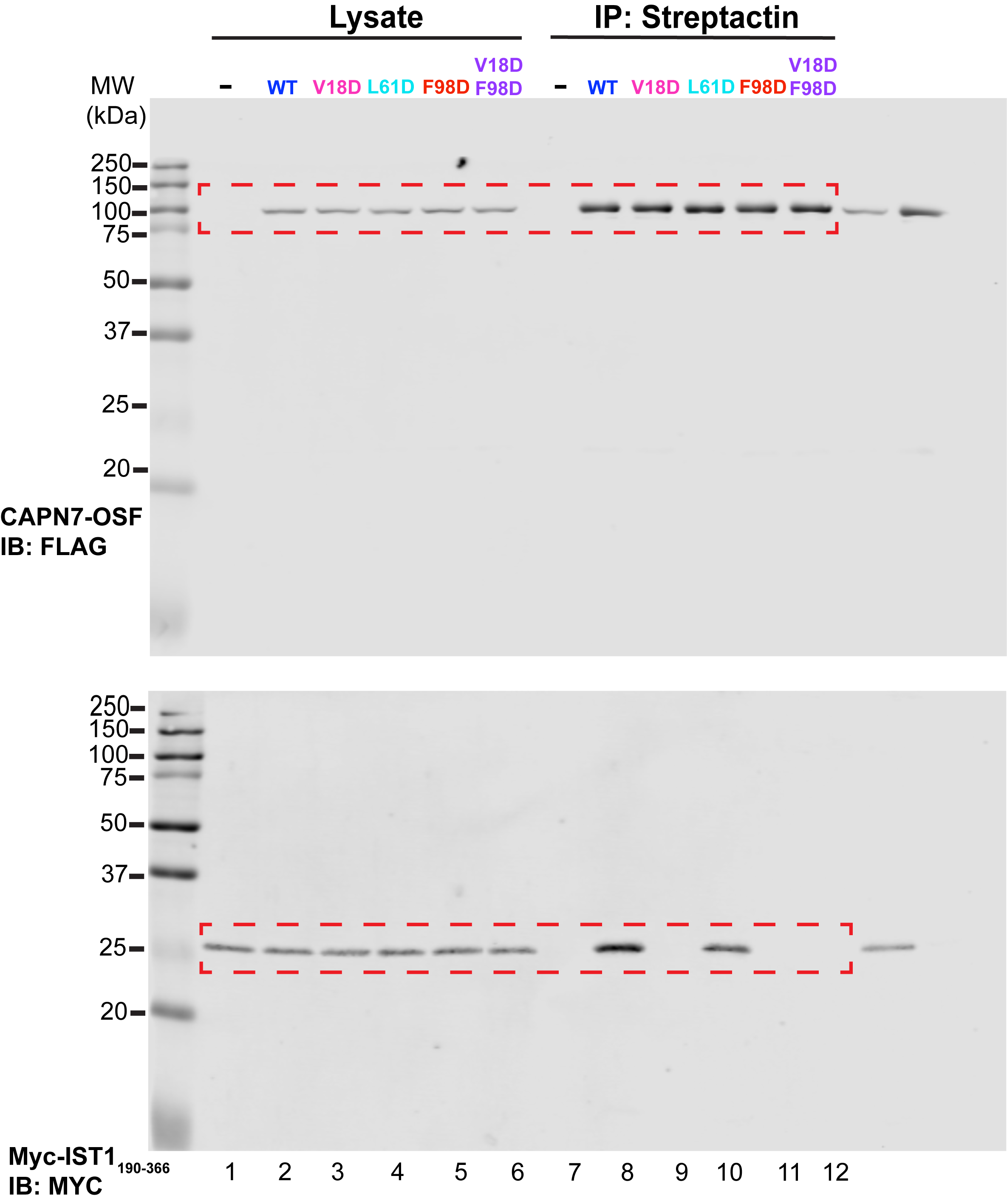

Supplement: Figure 3—source data 2. [file elife-84515-fig3-data2.zip › Figure 3-source data-2/Figure 3C-uncropped blots.tif]

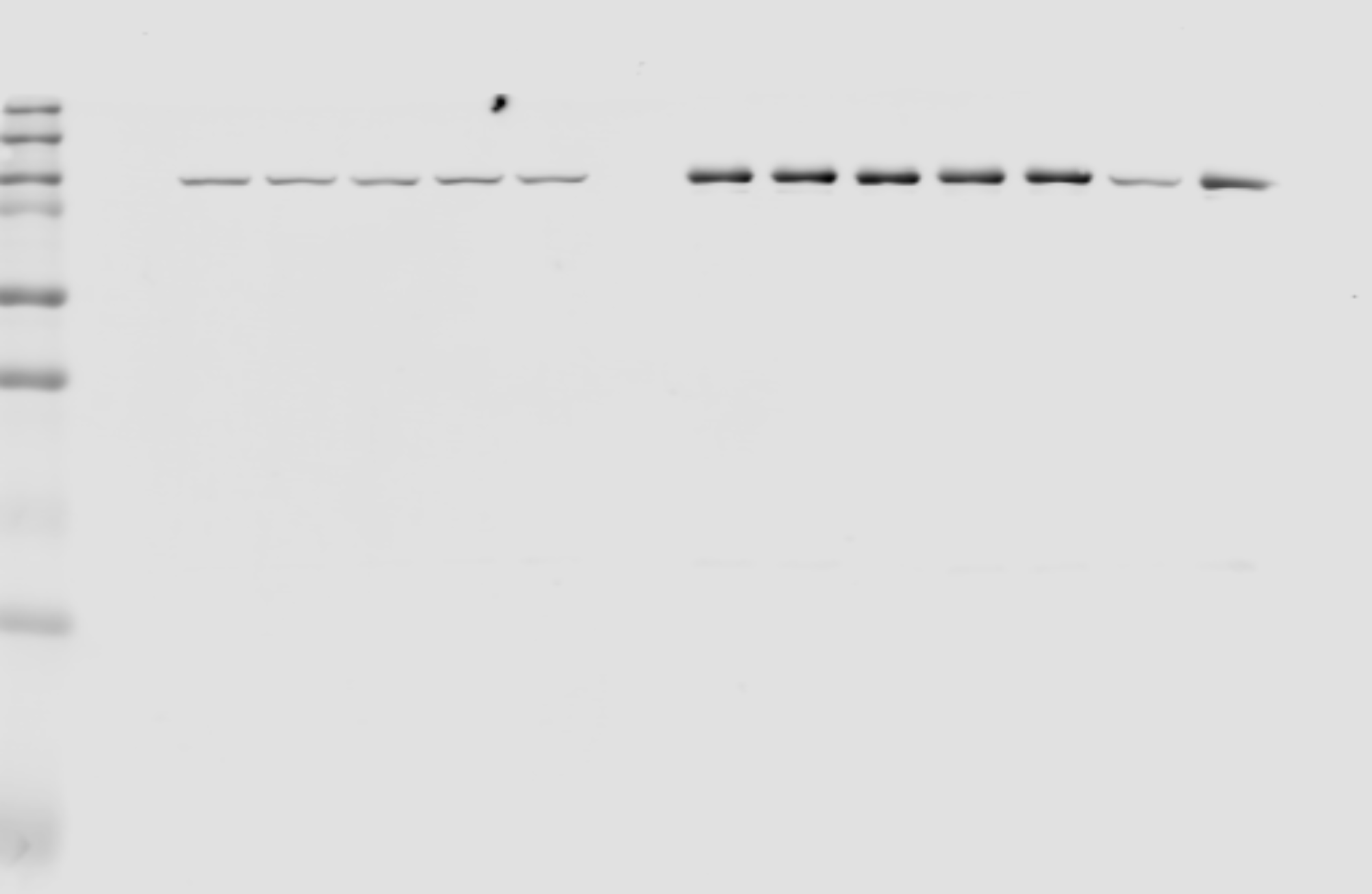

Supplement: Figure 3—source data 2. [file elife-84515-fig3-data2.zip › Figure 3-source data-2/Raw images/Figure 3C-antiFLAG.tif]

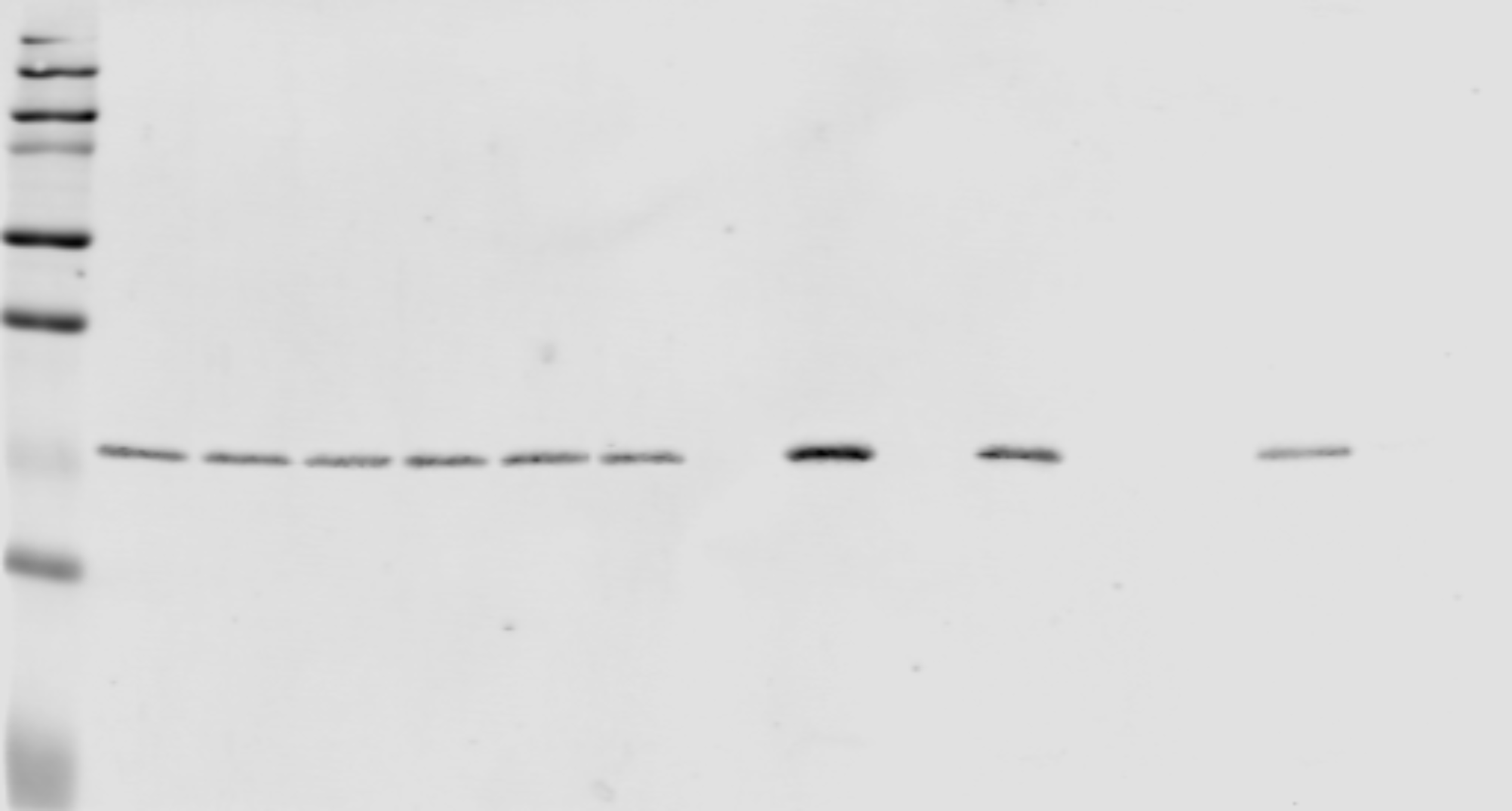

Supplement: Figure 3—source data 2. [file elife-84515-fig3-data2.zip › Figure 3-source data-2/Raw images/Figure 3C-antiMyc.tif]

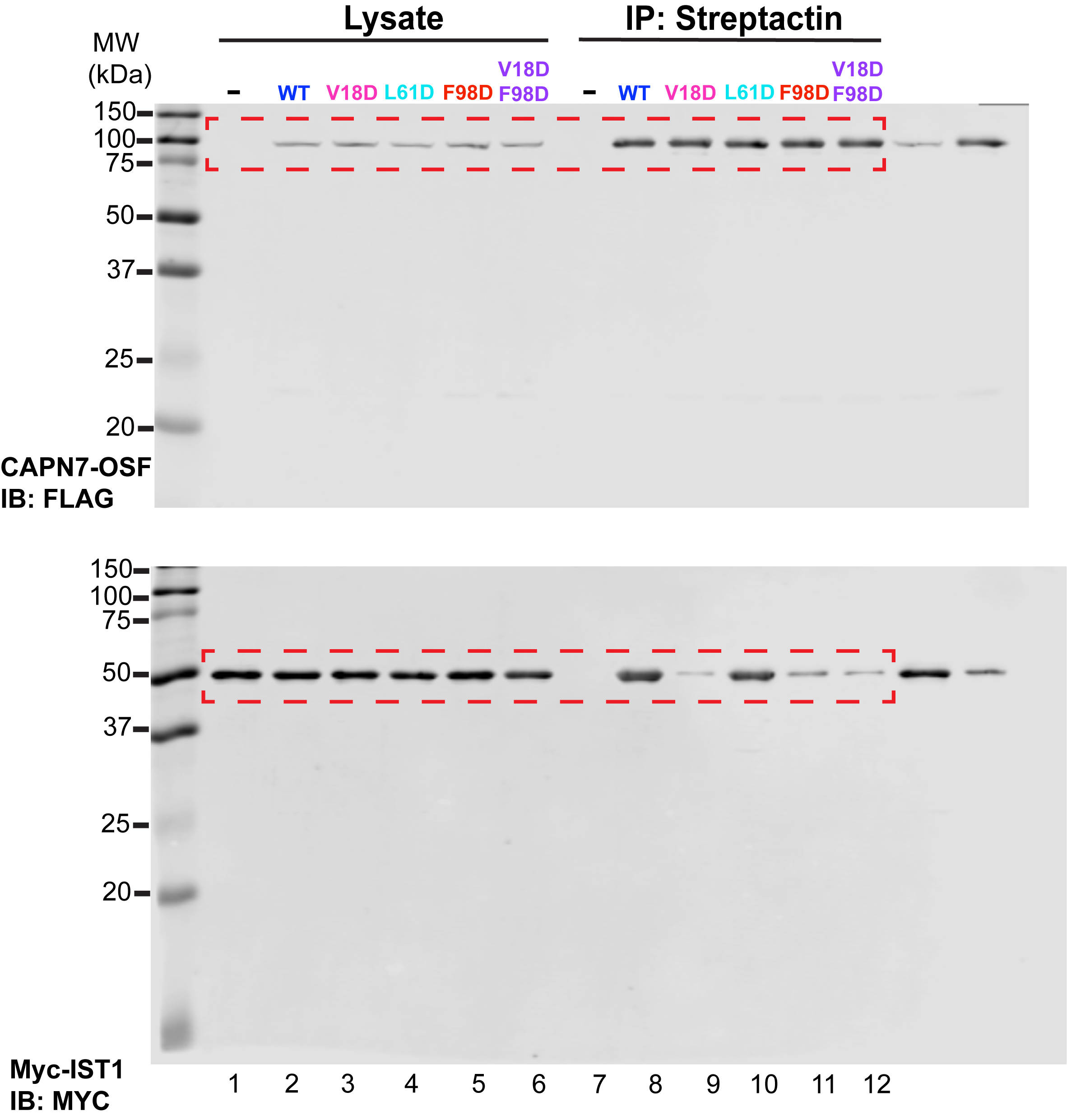

Supplement: Figure 3—figure supplement 1—source data 1. [file elife-84515-fig3-figsupp1-data1.zip › Figure 3-figure supplement 1-source data-1/Figure 3-supplement1B-uncropped blots.tif]

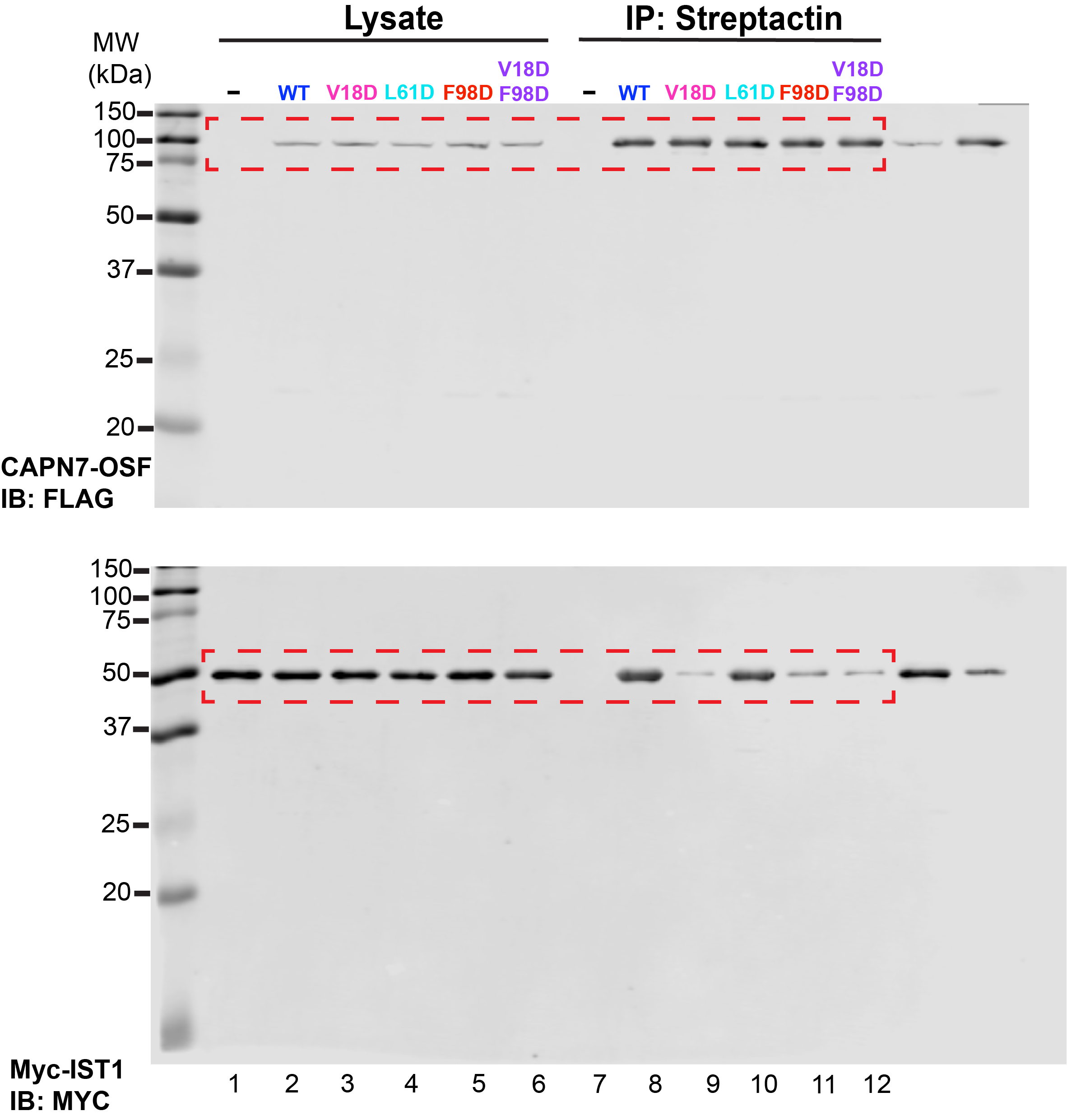

Supplement: Figure 3—figure supplement 1—source data 1. [file elife-84515-fig3-figsupp1-data1.zip › Figure 3-figure supplement 1-source data-1/Figure 3-supplement1B-uncropped blots.png]

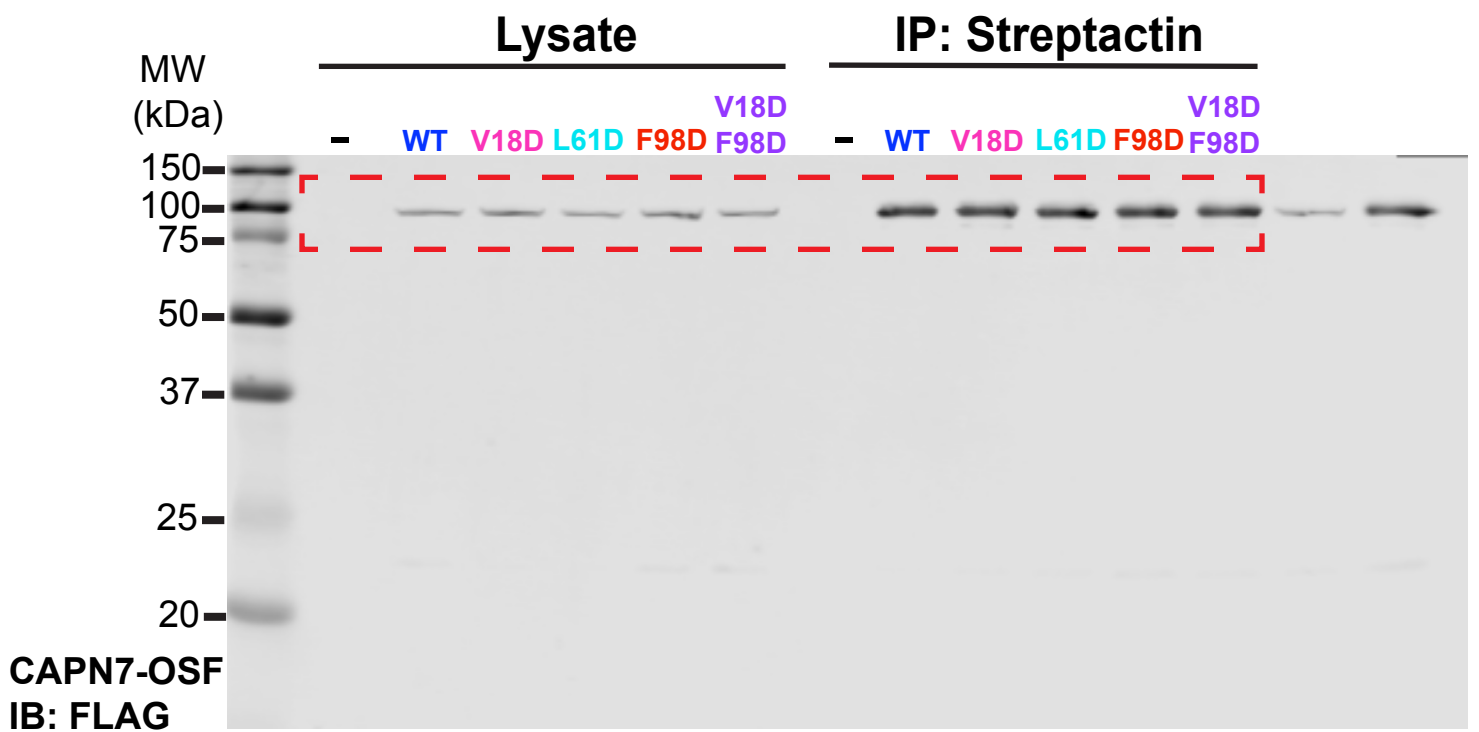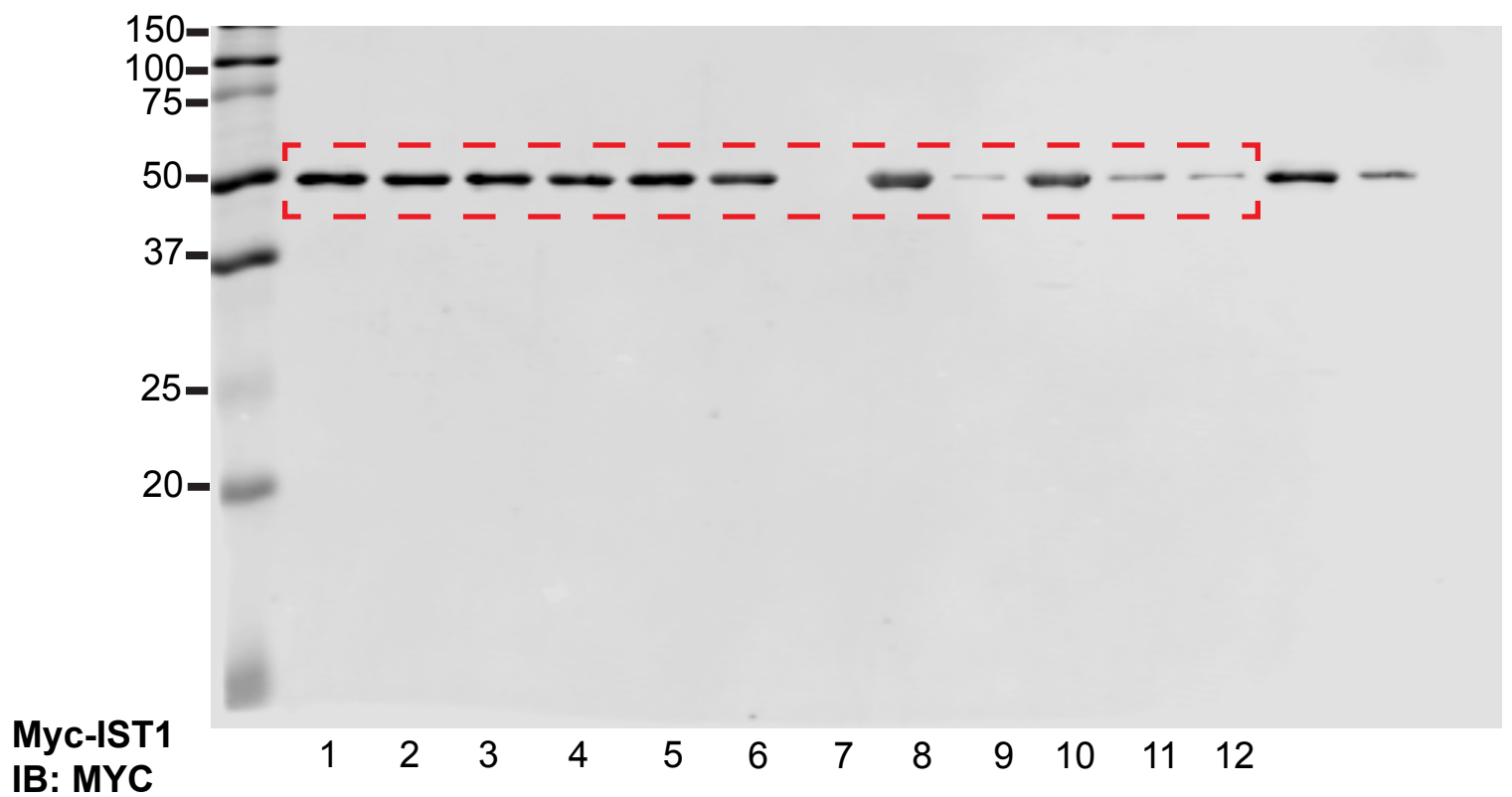

Supplement: Figure 3—figure supplement 1—source data 1. [file elife-84515-fig3-figsupp1-data1.zip › Figure 3-figure supplement 1-source data-1/Figure 3-supplement1B-uncropped blots.pdf]

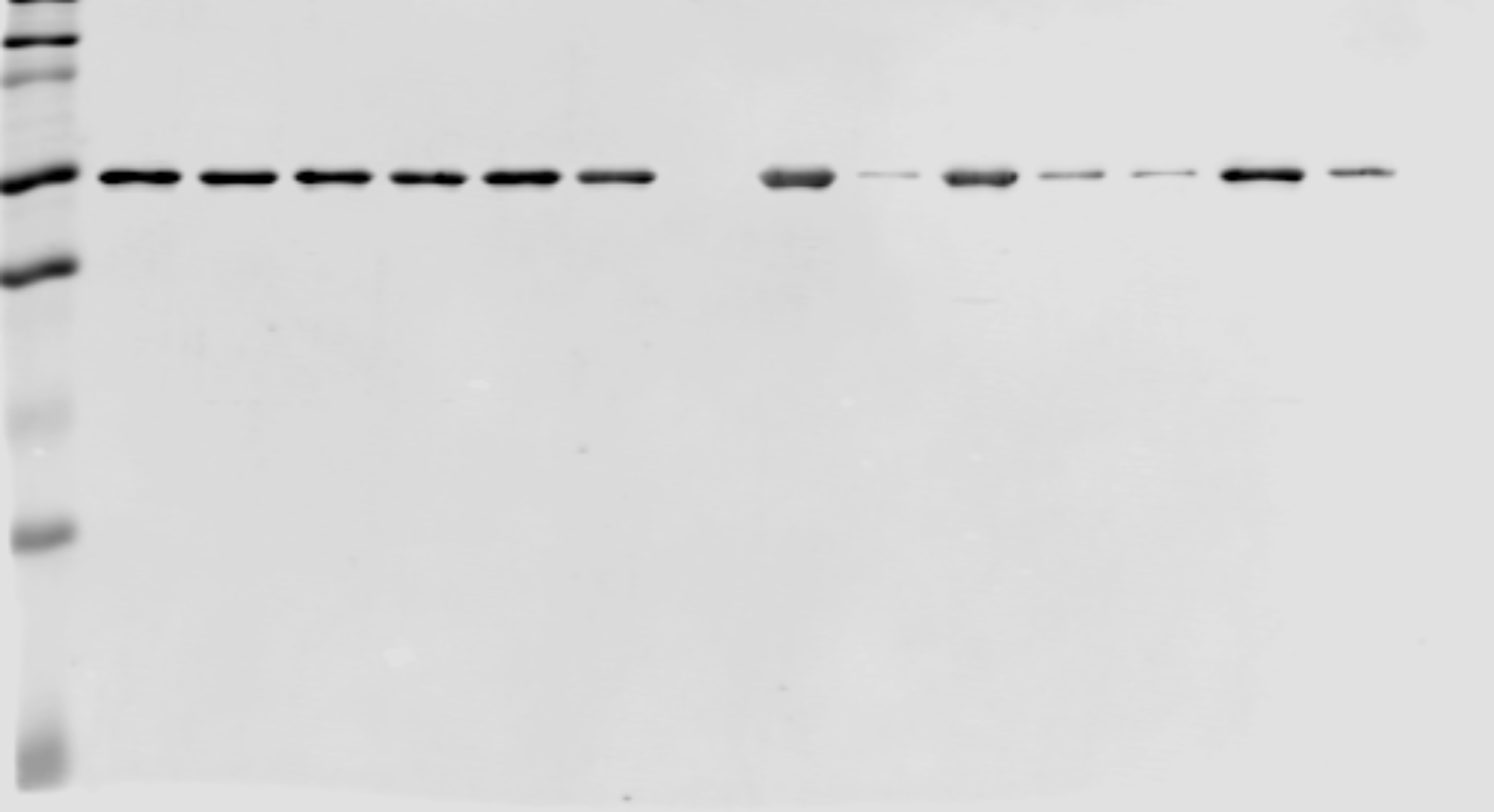

Supplement: Figure 3—figure supplement 1—source data 1. [file elife-84515-fig3-figsupp1-data1.zip › Figure 3-figure supplement 1-source data-1/Raw images/Figure 3-supplement 1B-antiMyc.tif]

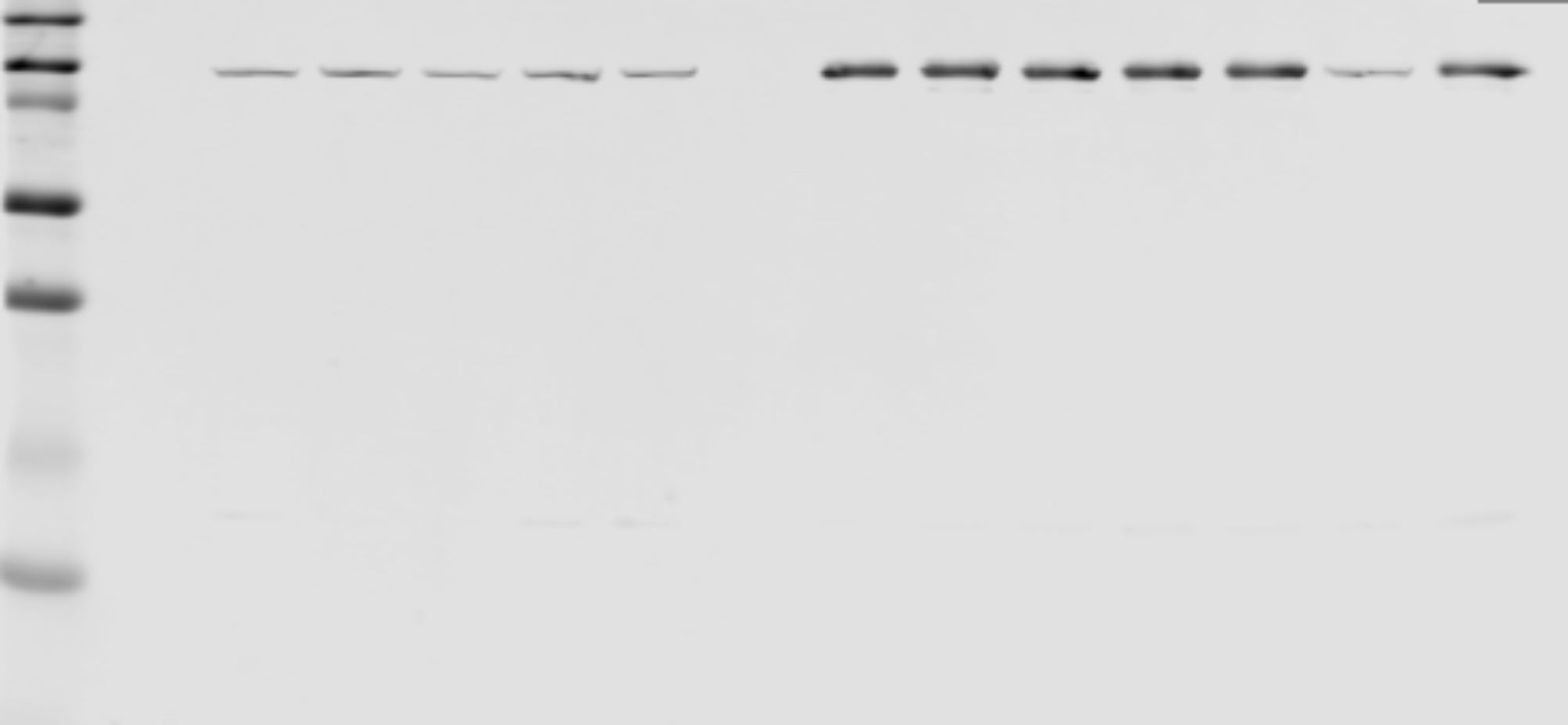

Supplement: Figure 3—figure supplement 1—source data 1. [file elife-84515-fig3-figsupp1-data1.zip › Figure 3-figure supplement 1-source data-1/Raw images/Figure 3-supplement 1B-antiFLAG.tif]

**siRNA 1:** NT ——— CAPN7 ———  
**siRNA 2:** NT ——— Nup153 ———  
**Rescue Construct:** mCh mCh WT V18D F98D C290S

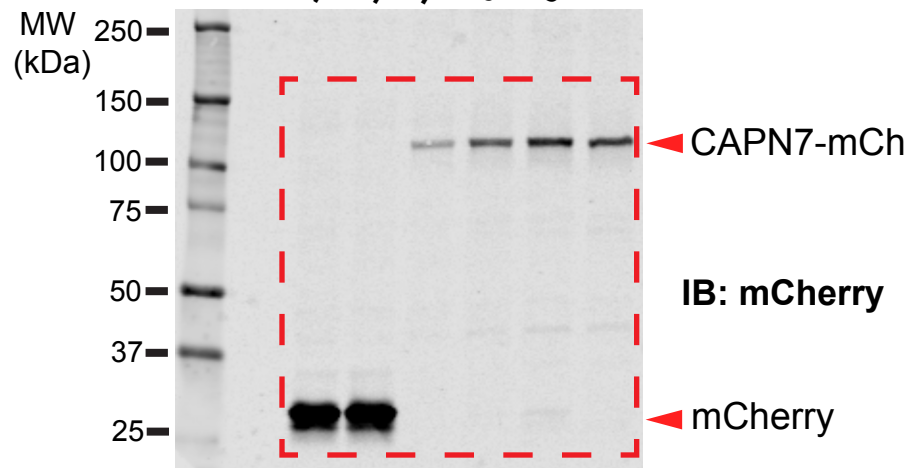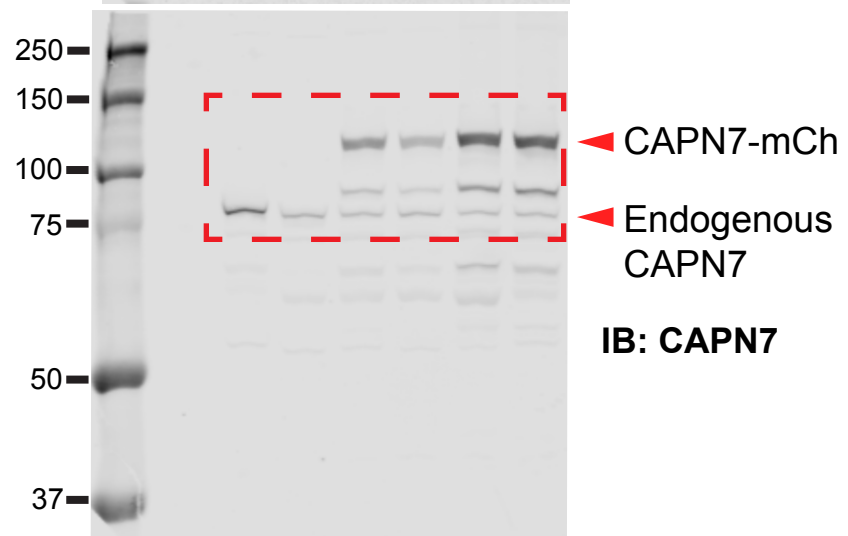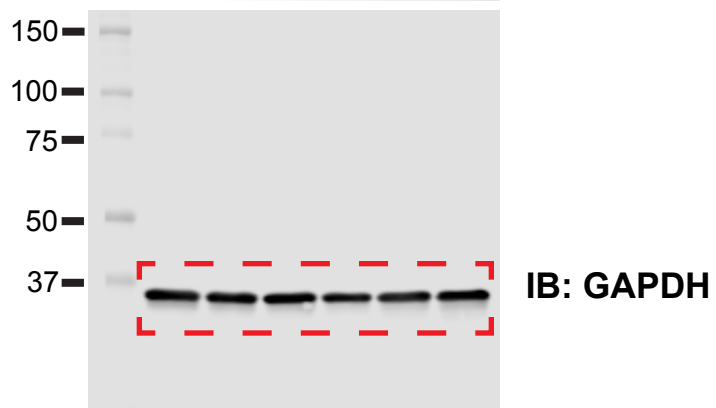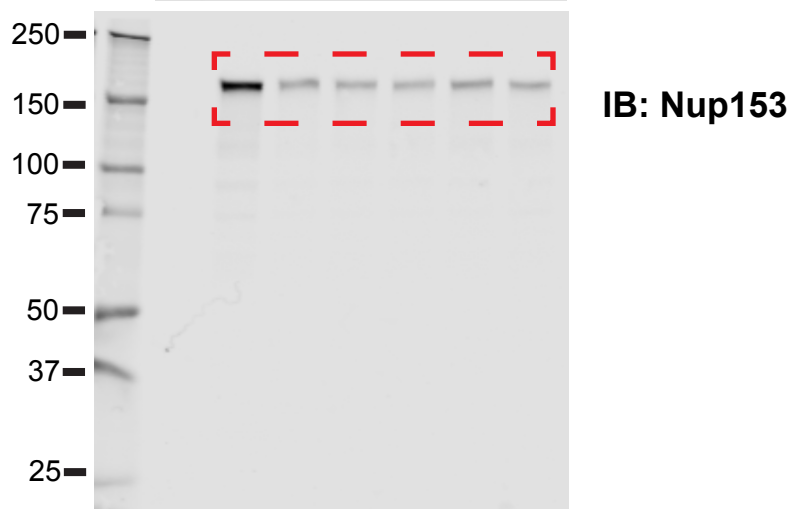

Supplement: Figure 4—figure supplement 1—source data 1. [file elife-84515-fig4-figsupp1-data1.zip › Figure 4-figure supplement 1-source data-1/Figure 4-supplement 1-uncropped blots.pdf]

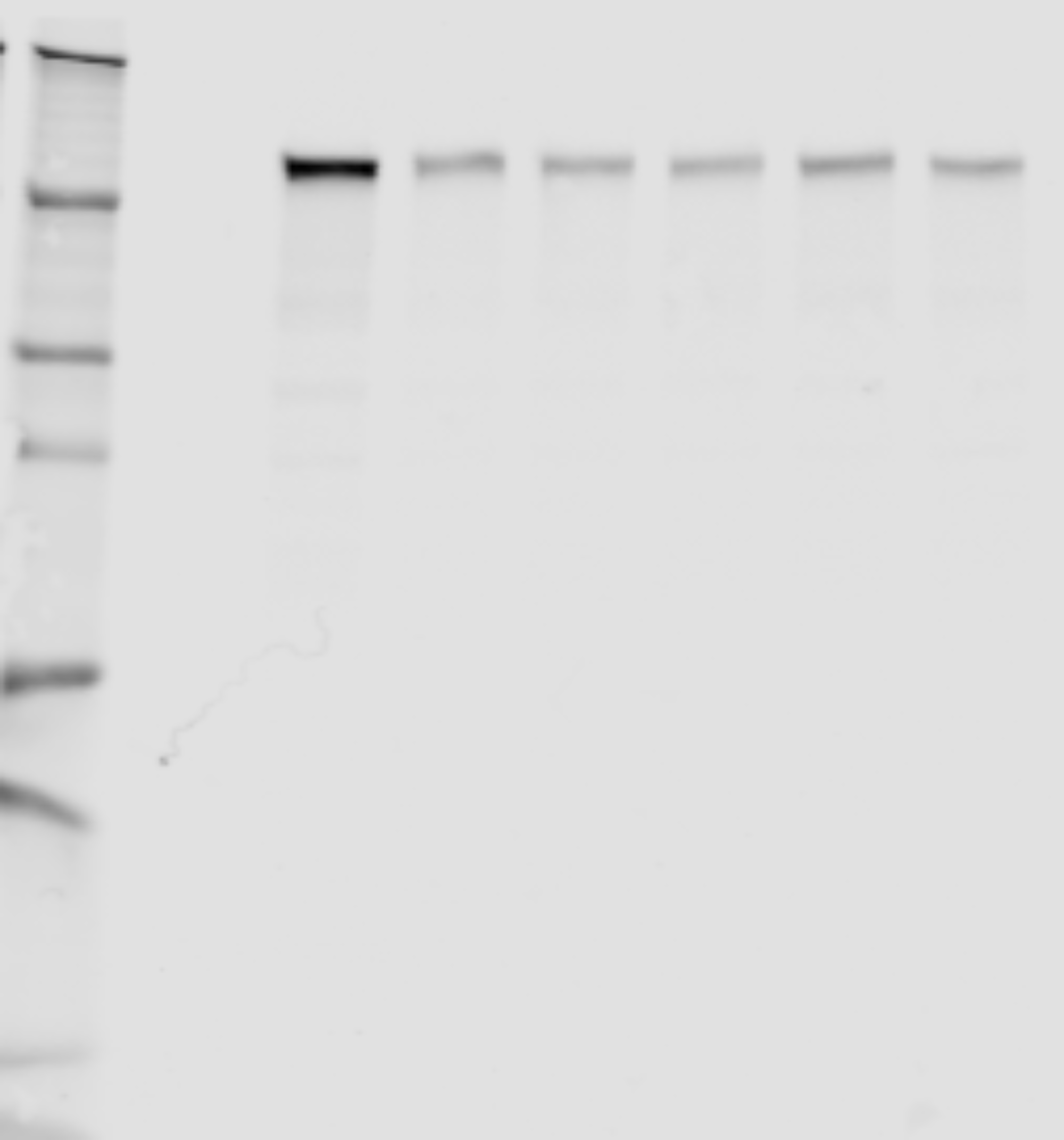

Supplement: Figure 4—figure supplement 1—source data 1. [file elife-84515-fig4-figsupp1-data1.zip › Figure 4-figure supplement 1-source data-1/Raw images/Figure 4-supplement 1-antiNup153.tif]

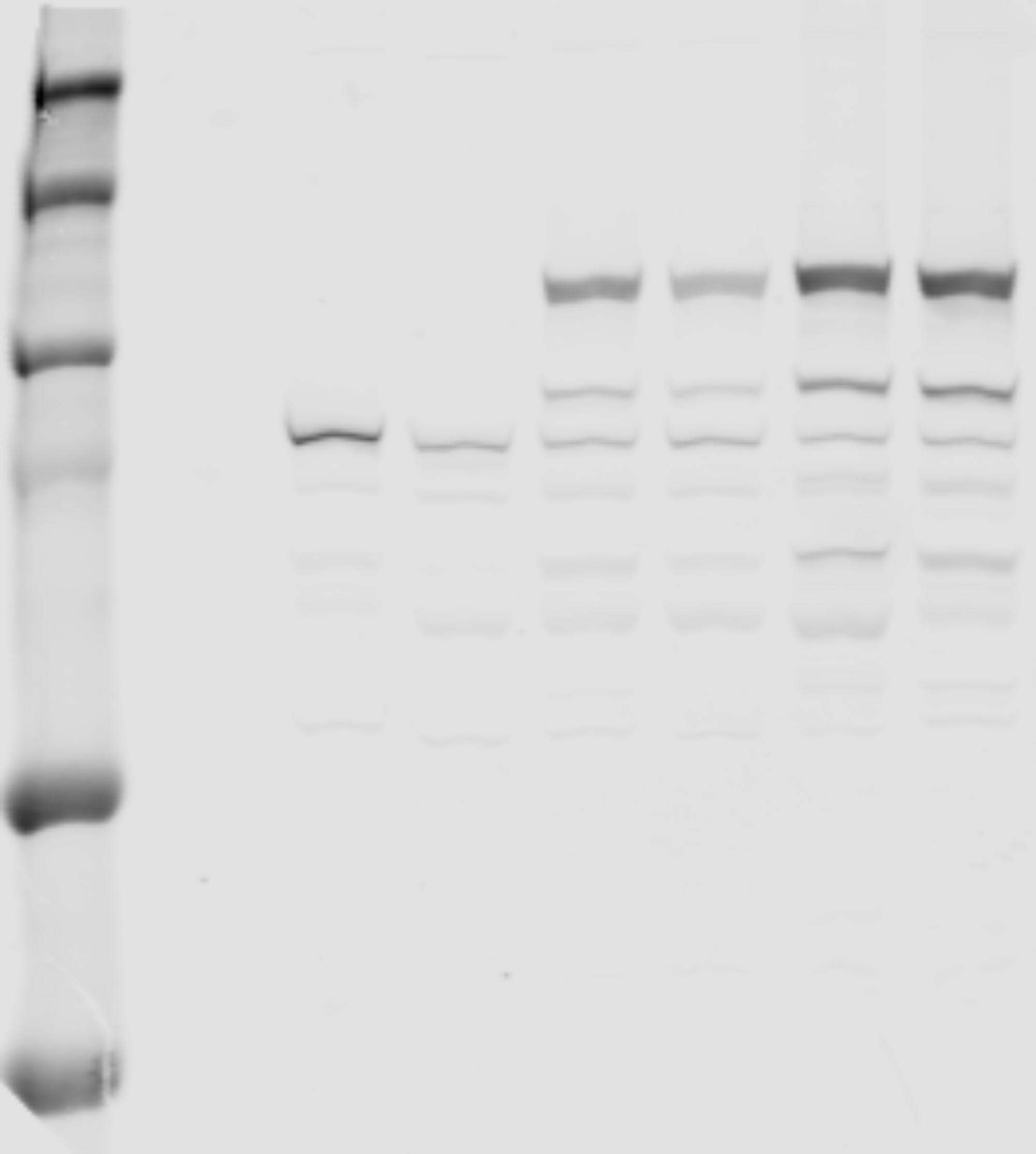

Supplement: Figure 4—figure supplement 1—source data 1. [file elife-84515-fig4-figsupp1-data1.zip › Figure 4-figure supplement 1-source data-1/Raw images/Figure 4-supplement 1-antiCAPN7.tif]

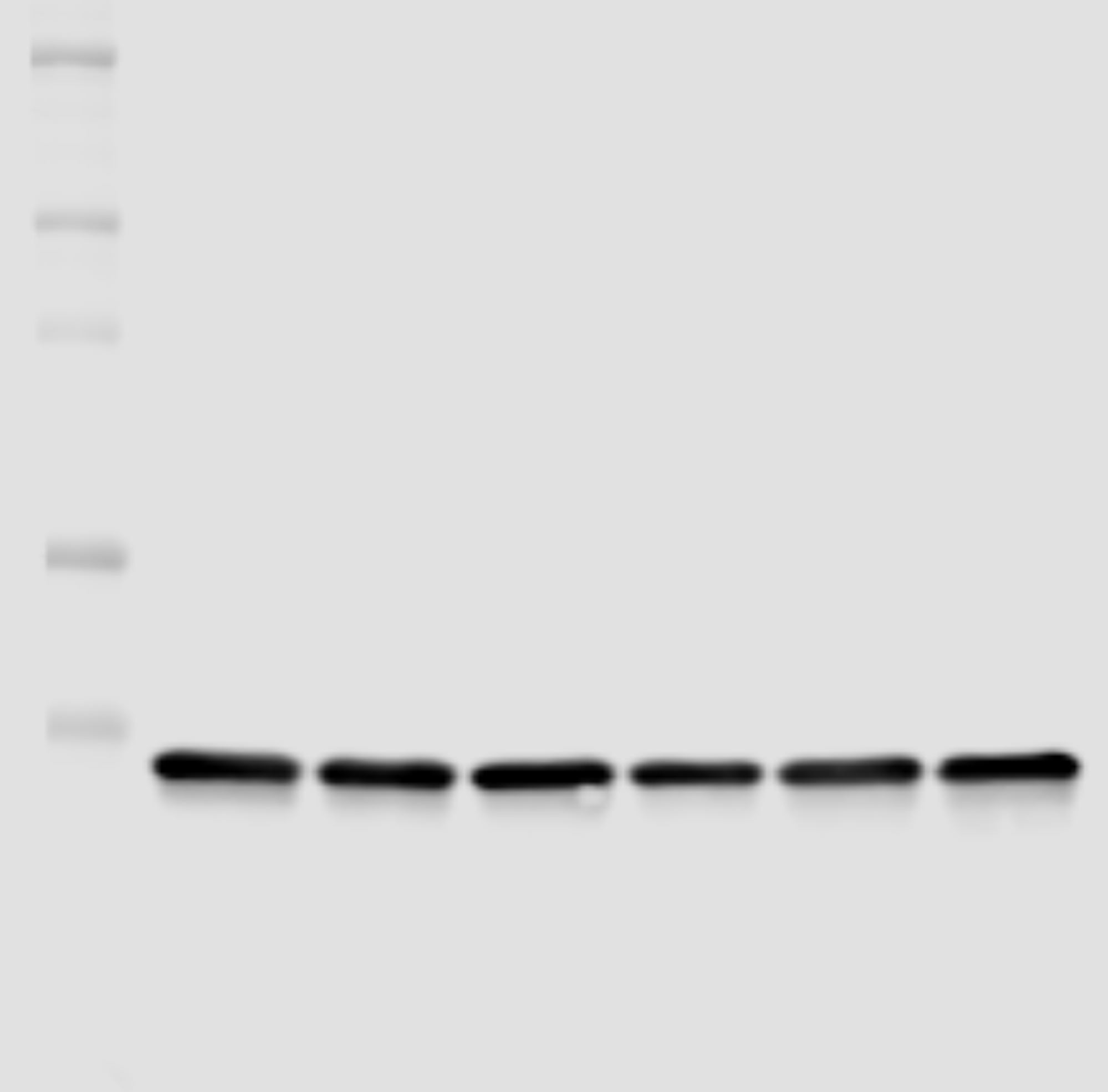

Supplement: Figure 4—figure supplement 1—source data 1. [file elife-84515-fig4-figsupp1-data1.zip › Figure 4-figure supplement 1-source data-1/Raw images/Figure 4-supplement 1-antiGAPDH.tif]

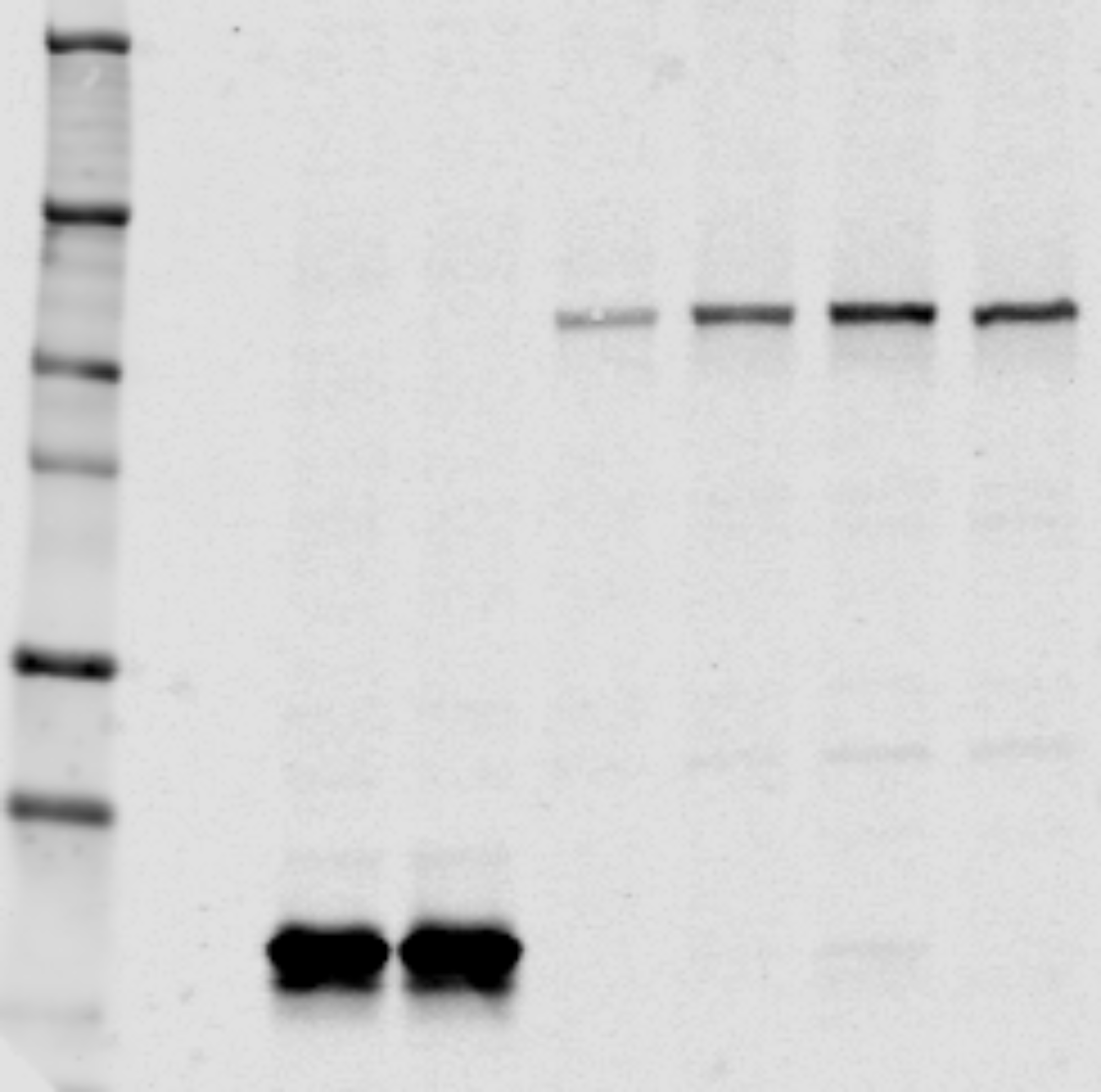

Supplement: Figure 4—figure supplement 1—source data 1. [file elife-84515-fig4-figsupp1-data1.zip › Figure 4-figure supplement 1-source data-1/Raw images/Figure 4-supplement 1-antimCherry.tif]

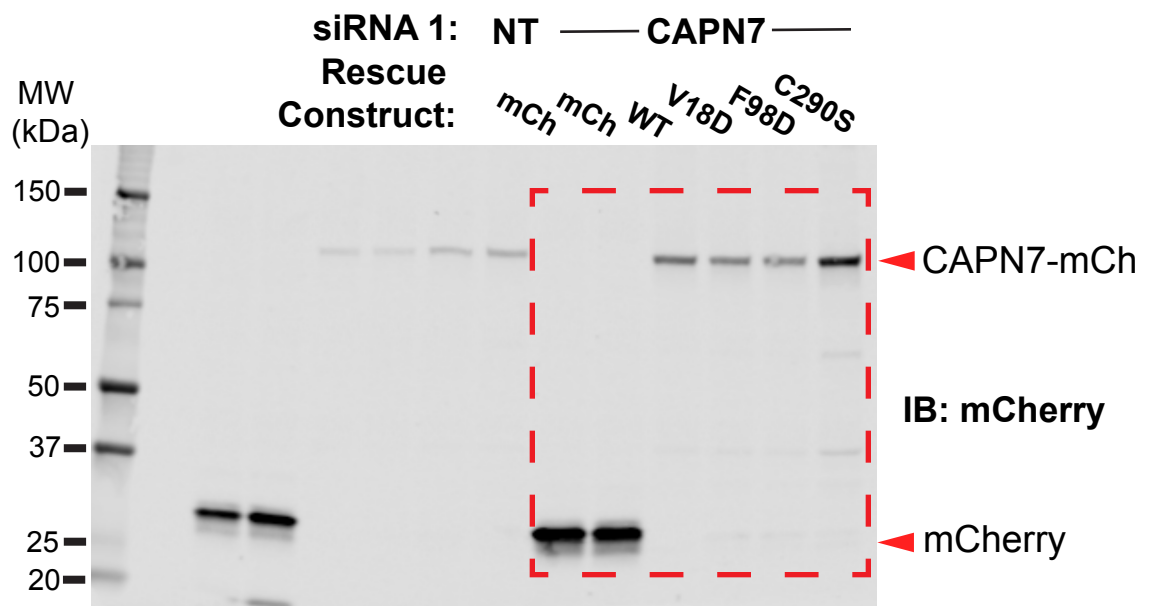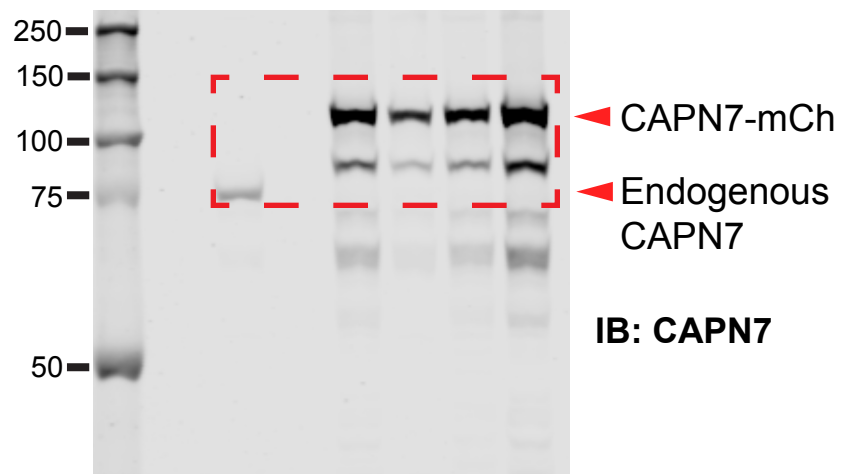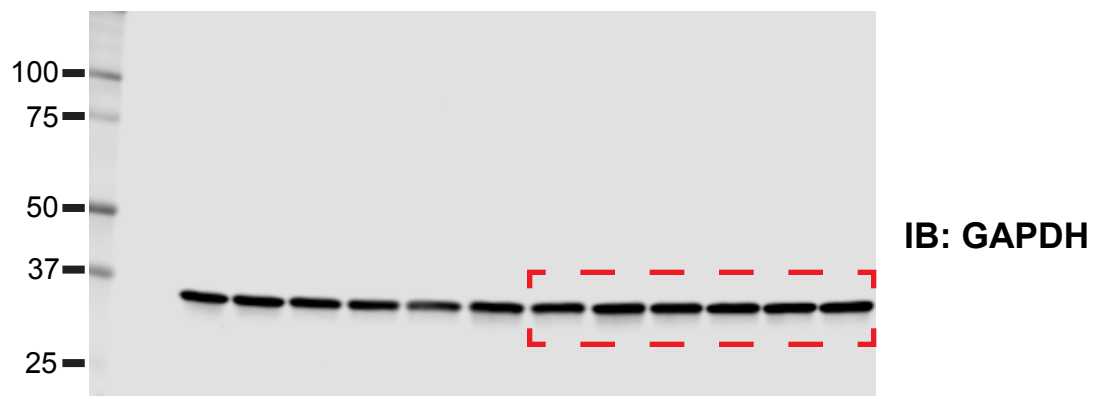

Supplement: Figure 5—figure supplement 1—source data 1. [file elife-84515-fig5-figsupp1-data1.zip › Figure 5-figure supplement 1-source data-1/Figure 5-supplement1A-uncropped blots.pdf]

siRNA 1: NT — CAPN7 —

siRNA 2: NT — Nup153 —

Rescue  
Construct: mCh mCh WT V18D F98D C290S

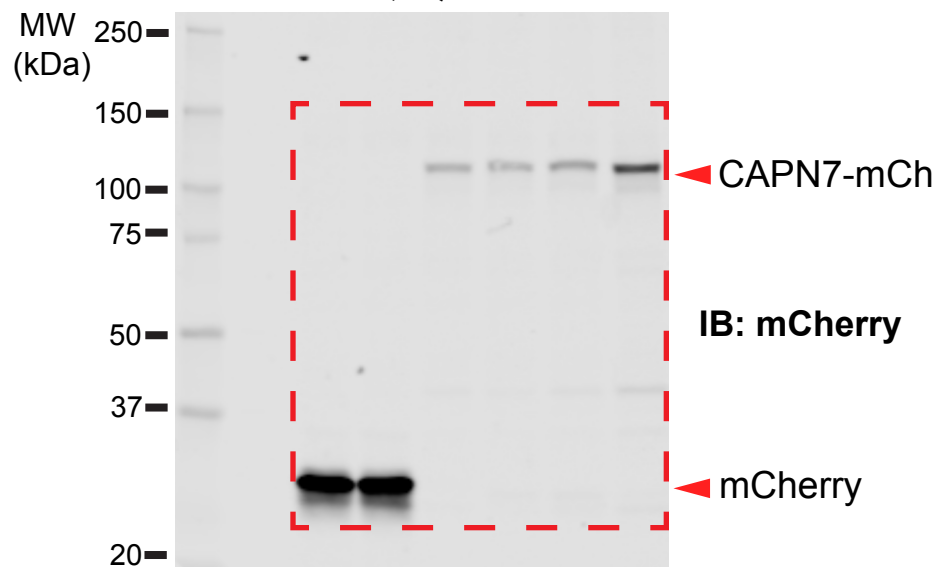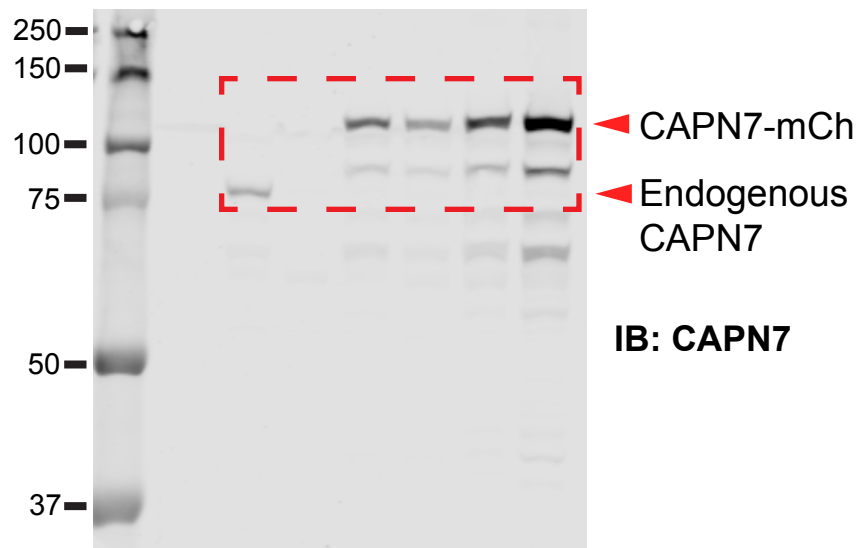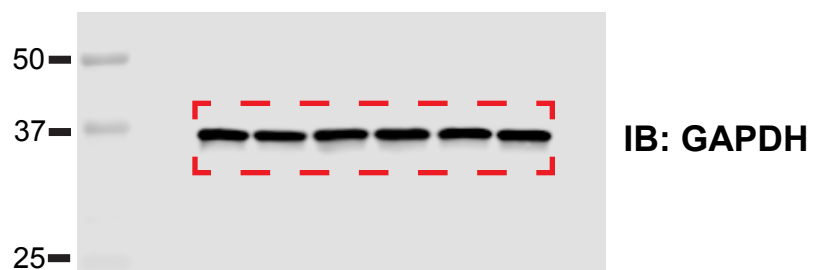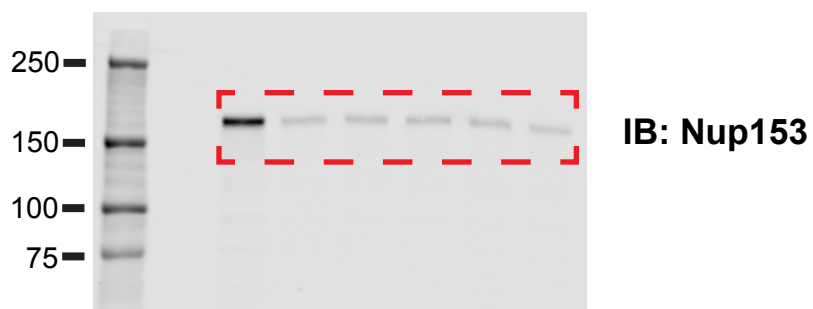

Supplement: Figure 5—figure supplement 1—source data 1. [file elife-84515-fig5-figsupp1-data1.zip › Figure 5-figure supplement 1-source data-1/Figure 5-supplement1B-uncropped blots.pdf]

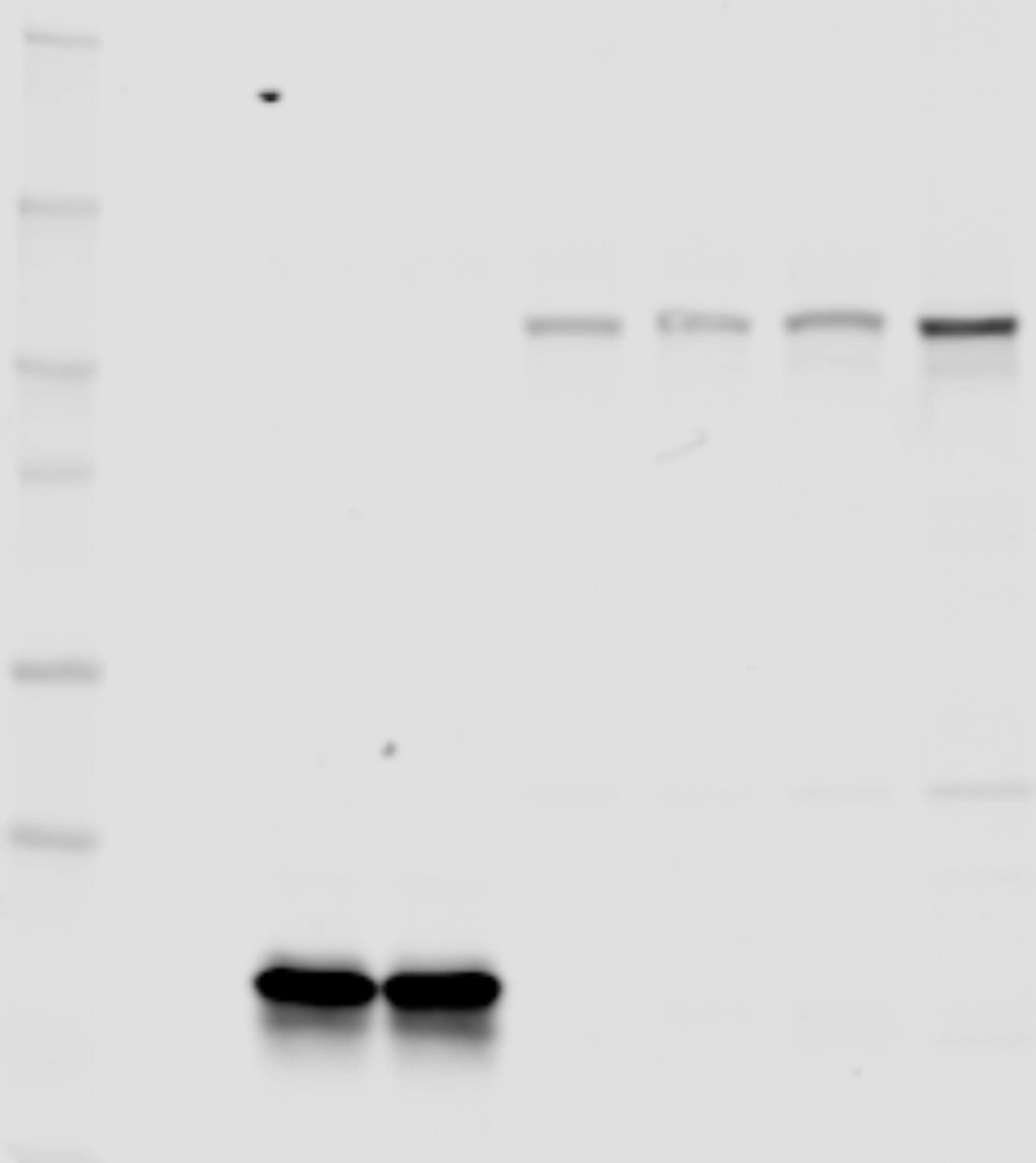

Supplement: Figure 5—figure supplement 1—source data 1. [file elife-84515-fig5-figsupp1-data1.zip › Figure 5-figure supplement 1-source data-1/Raw images/Figure 5-supplement 1B-antimCherry.tif]

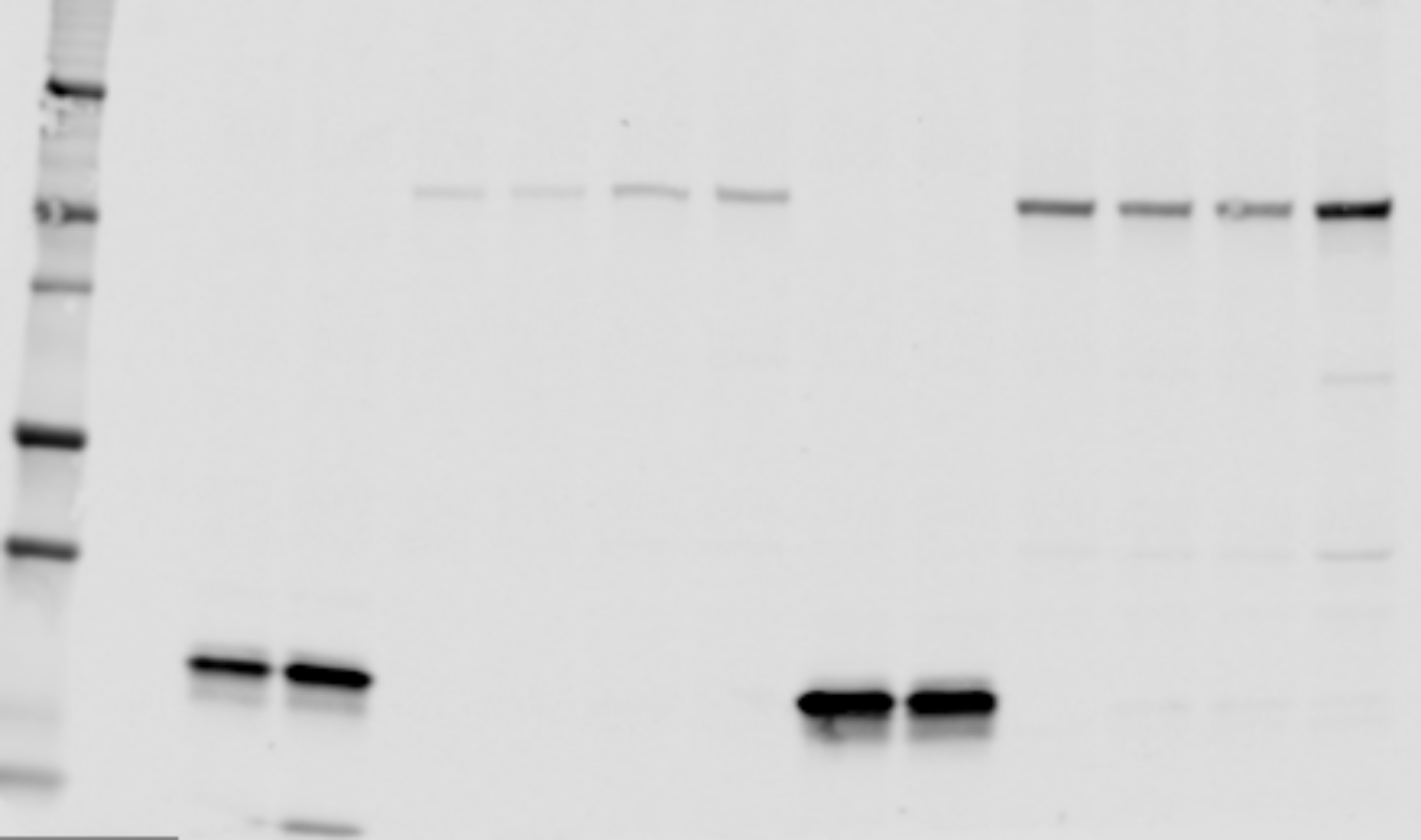

Supplement: Figure 5—figure supplement 1—source data 1. [file elife-84515-fig5-figsupp1-data1.zip › Figure 5-figure supplement 1-source data-1/Raw images/Figure 5-supplement 1A-antimCherry.tif]

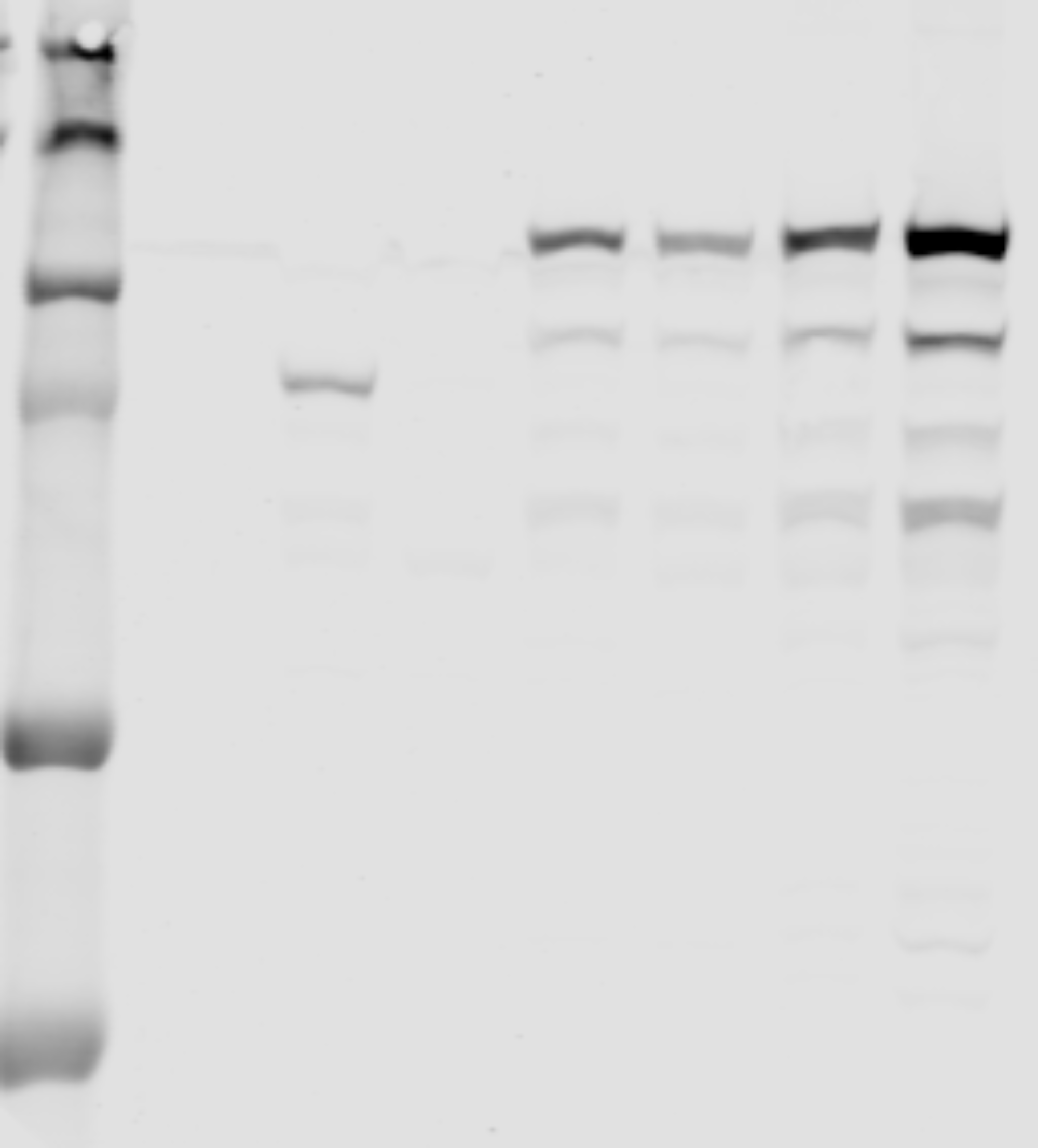

Supplement: Figure 5—figure supplement 1—source data 1. [file elife-84515-fig5-figsupp1-data1.zip › Figure 5-figure supplement 1-source data-1/Raw images/Figure 5-supplement 1B-antiCAPN7.tif]

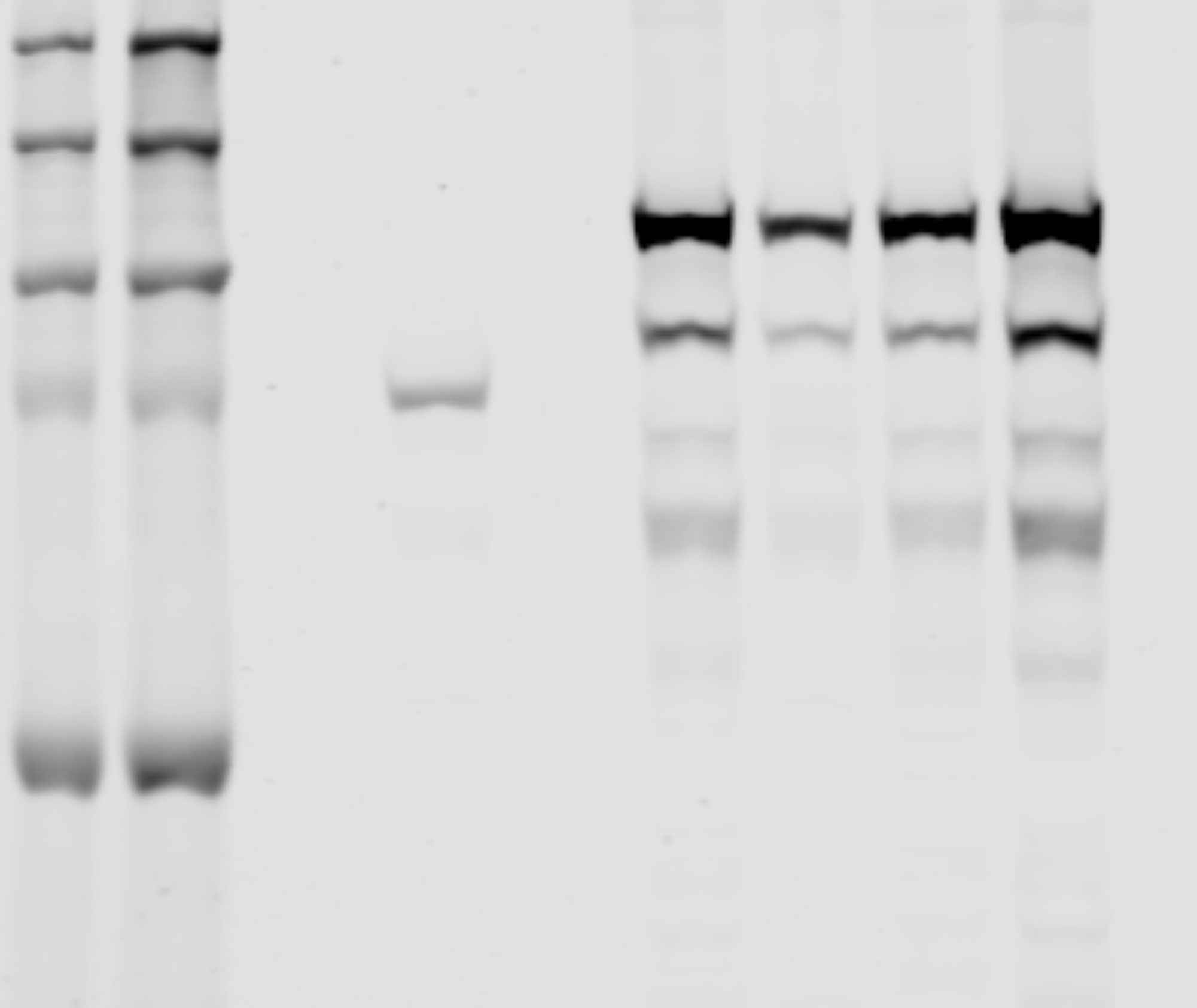

Supplement: Figure 5—figure supplement 1—source data 1. [file elife-84515-fig5-figsupp1-data1.zip › Figure 5-figure supplement 1-source data-1/Raw images/Figure 5-supplement 1A-antiCAPN7.tif]

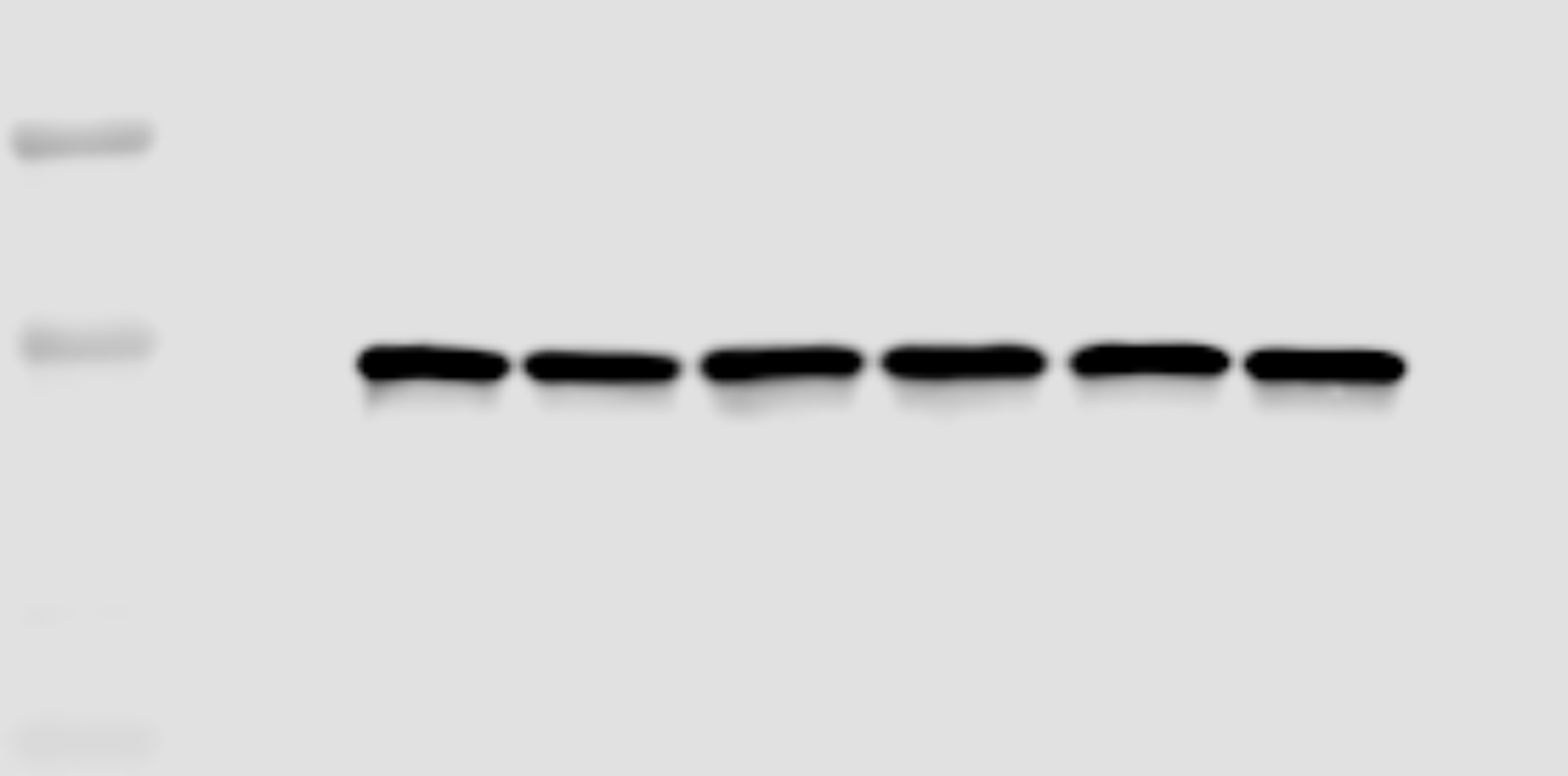

Supplement: Figure 5—figure supplement 1—source data 1. [file elife-84515-fig5-figsupp1-data1.zip › Figure 5-figure supplement 1-source data-1/Raw images/Figure 5-supplement 1B-antiGAPDH.tif]

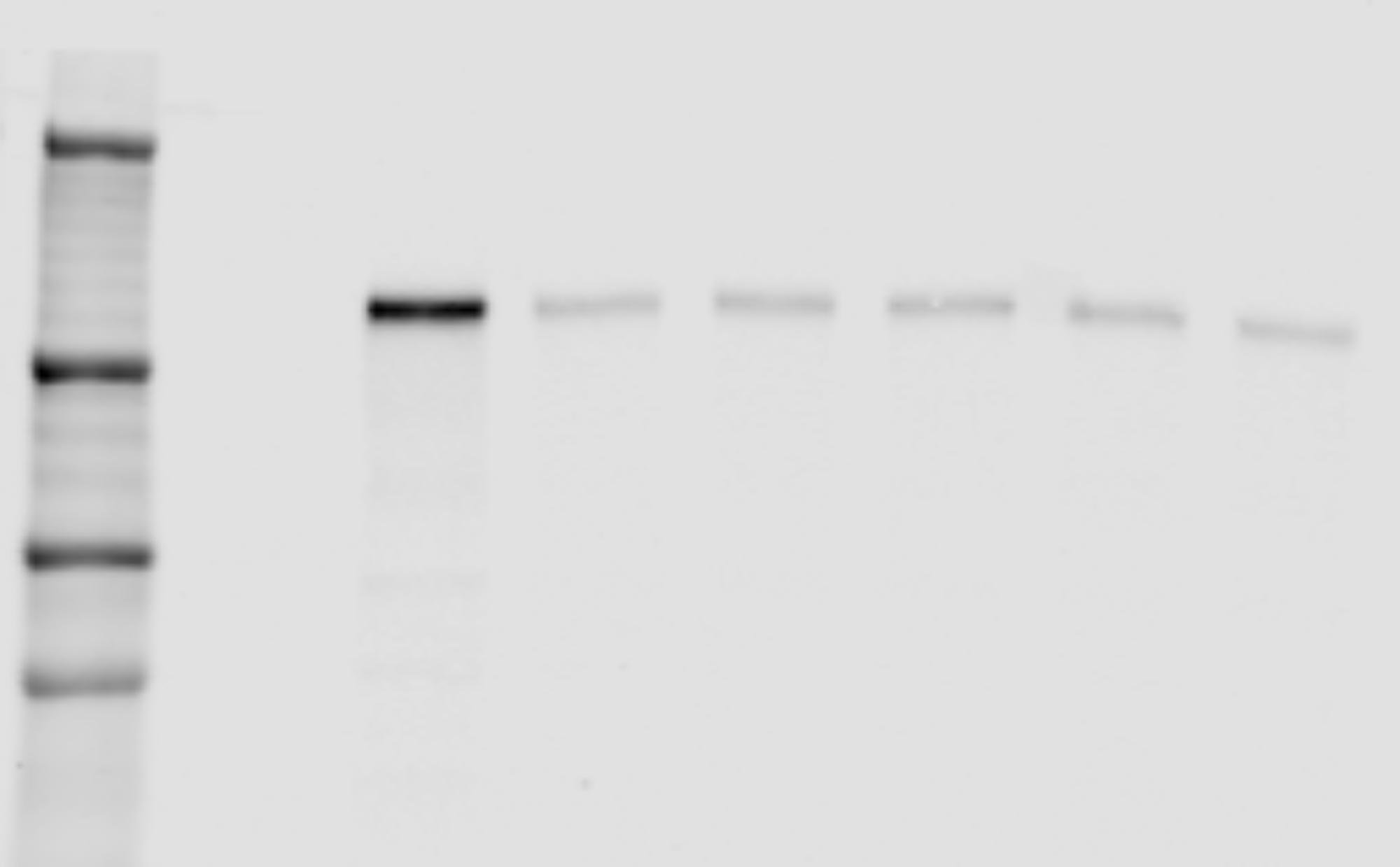

Supplement: Figure 5—figure supplement 1—source data 1. [file elife-84515-fig5-figsupp1-data1.zip › Figure 5-figure supplement 1-source data-1/Raw images/Figure 5-supplement 1B-antiNup153.tif]

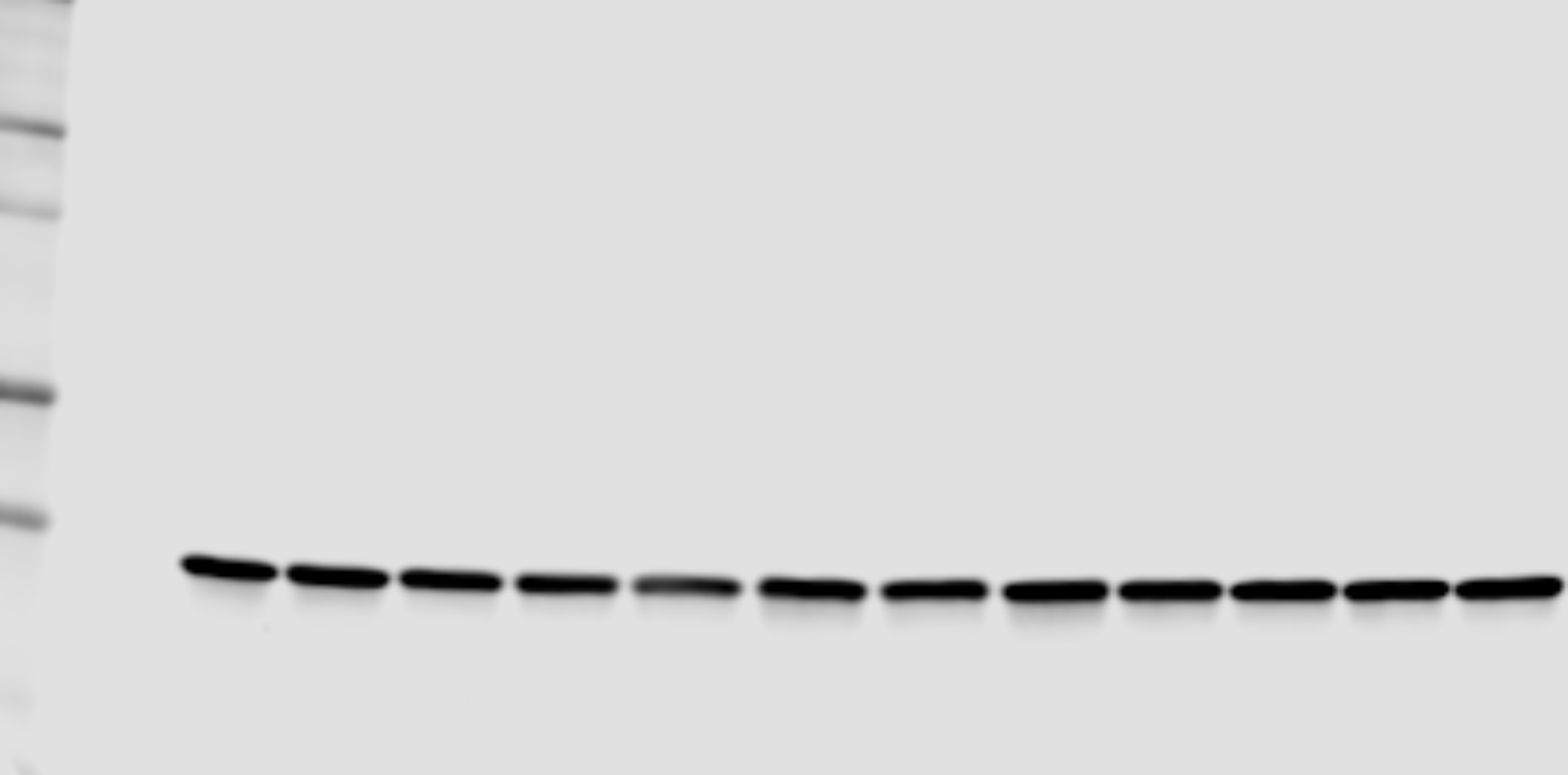

Supplement: Figure 5—figure supplement 1—source data 1. [file elife-84515-fig5-figsupp1-data1.zip › Figure 5-figure supplement 1-source data-1/Raw images/Figure 5-supplement 1A-antiGAPDH.tif]
