# Supplementary material for: The Calpain-7 protease functions together with the ESCRT-III protein IST1 within the midbody to regulate the timing and completion of abscission
Source: eLife. 2023 Sep 29;12:e84515. doi: 10.7554/eLife.84515 (PMC10586806; doi:10.7554/eLife.84515)
Supplement: Supplementary file 1. [file elife-84515-supp1.docx]

**Supplementary File 1. ESI-MS mass confirmation of purified proteins.**

| **Expression plasmid** | **Expected Mass (Da)** | **Measured Mass (Da)** | **Uniprot #** | **Notes** |
| --- | --- | --- | --- | --- |
| pCA528 CAPN7 (1-165) | 18579.80 | 18581.34 | Q9Y6W3 | Wenzel *et al.,* 2022 |
| pCA528 CAPN7 V18D (1-165) | 18595.75 | 18595.35 | Q9Y6W3 | Wenzel *et al.,* 2022 |
| pCA528 CAPN7 L61D (1-165) | 18581.73 | 18581.35 | Q9Y6W3 |  |
| pCA528 CAPN7 F98D (1-165) | 18547.41 | 18547.35 | Q9Y6W3 | Wenzel *et al.,* 2022 |
| pCA528 CAPN7 V18D, F98D (1-165) | 18562.31 | 18562.37 | Q9Y6W3 |  |
| pCA528 CAPN7 (1-813) | 92652.41 | 92650.89 | Q9Y6W3 |  |
| pCA528 IST1 (316-366) (C-Cys) | 6252.69 | 6251.69 | P53390-4 | Non-native N-terminal Gly, Non-native C-terminal GlyCys reacted with Oregon Green. Wenzel *et al.,* 2022 |
| pCA528 IST1 L328D (316-366)(C-Cys) | 6253.76 | 6253.73 | P53390-4 | Non-native N-terminal Gly, Non-native C-terminal GlyCys reacted with Oregon Green. |
| pCA528 IST1 L355A (316-366)(C-Cys) | 6209.77 | 6210.74 | P53390-4 | Non-native N-terminal Gly, Non-native C-terminal GlyCys reacted with Oregon Green. |
| pCA528 IST1 L328D,L355A (316-366)(C-Cys) | 6211.71 | 6211.70 | P53390-4 | Non-native N-terminal Gly, Non-native C-terminal GlyCys reacted with Oregon Green. |
| pCA528 IST1 (322-366) | 4895.46 | 4895.45 | P53390-4 |  |
| pCA528 IST1 (1-366) | 39978.83 | 39978.54 | P53390-4 |  |
| pCA528 IST1 L328D, L355A (1-366) | 39937.51 | 39937.33 | P53390-4 |  |
|  |  |  |  |  |
