# Supplementary material for: The Calpain-7 protease functions together with the ESCRT-III protein IST1 within the midbody to regulate the timing and completion of abscission
Source: eLife. 2023 Sep 29;12:e84515. doi: 10.7554/eLife.84515 (PMC10586806; doi:10.7554/eLife.84515)
Supplement: Supplementary file 2. [file elife-84515-supp2.docx]

**Supplementary File 2. Plasmids**

| **Plasmid** | **Internal ID** | **Addgene or DNASU #** | **Uniprot #** | **Source/**  **Reference** |
| --- | --- | --- | --- | --- |
| **MIT DOMAINS** |  |  |  |  |
| pCA528 CAPN7 (1-75) | WISP22-30 | 193031 (Addgene) | Q9Y6W3 | This study |
| pCA528 CAPN7 (81-165) | WISP22-31 | 193032 (Addgene) | Q9Y6W3 | This study |
| pCA528 CAPN7 (1-165) | WISP21-25 | 180611 (Addgene) | Q9Y6W3 | Wenzel *et al.,* 2022 |
| pCA528 CAPN7 V18D (1-165) | WISP21-26 | 180612 (Addgene) | Q9Y6W3 | Wenzel *et al.,* 2022 |
| pCA528 CAPN7 L61D (1-165) | WISP22-29 | 193033 (Addgene) | Q9Y6W3 | This study |
| pCA528 CAPN7 F98D (1-165) | WISP21-27 | 180613 (Addgene) | Q9Y6W3 | Wenzel *et al.,* 2022 |
| pCA528 CAPN7 V18D,F98D (1-165) | WISP22-32 | 193034 (Addgene) | Q9Y6W3 | This Study |
|  |  |  |  |  |
| **ESCRT-III Peptides** |  |  |  |  |
| pGEX IST1 (303-366) | WISP07-210 |  | P53390-4 | Bajorek *et al.,* 2009 |
| pCA528 IST1 (316-366) (C-Cys) | WISP20-100 |  | P53390-4 | Wenzel *et al.,* 2022 |
| pCA528 IST1 L328D (316-366)(C-Cys) | WISP22-33 | 193035 (Addgene) | P53390-4 | This study |
| pCA528 IST1 L355A (316-366)(C-Cys) | WISP22-34 | 193036 (Addgene) | P53390-4 | This study |
| pCA528 IST1 L328D,L355A (316-366)(C-Cys) | WISP22-35 | 193037 (Addgene) | P53390-4 | This study |
| pCA528 IST1 (322-366) | WISP22-36 | 193038 (Addgene) | P53390-4 | This study |
| pCA528 IST1 (1-366) | WISP20-12 | 193039 (Addgene) | P53390-4 | This study |
| pCA528 IST1 L328D, L355A (1-366) | WISP22-37 | 193040 (Addgene) | P53390-4 | This study |
|  |  |  |  |  |
| **Mammalian Expression Vectors** |  |  |  |  |
| pCAG-OSF (MCS2) | WISP08-103 |  |  | Morita *et al.,* 2010 / Robert A. Lamb gift |
| pCAG CAPN7-OSF | WISP22-38 | 193041 (Addgene) | Q9Y6W3 | This study |
| pCAG CAPN7 V18D-OSF | WISP22-39 | 193042 (Addgene) | Q9Y6W3 | This study |
| pCAG CAPN7 L61D-OSF | WISP22-40 | 193043 (Addgene) | Q9Y6W3 | This study |
| pCAG CAPN7 F98D-OSF | WISP22-41 | 193044 (Addgene) | Q9Y6W3 | This study |
| pCAG CAPN7 V18D,F98D-OSF | WISP22-42 | 193045 (Addgene) | Q9Y6W3 | This study |
| pCAG Myc-IST1 | WISP07-77 | HSCD00751713 (DNASU) | P53390 | Bajorek *et al.,* 2009 |
| pCAG Myc-IST1 (190-366) | WISP08-20 |  | P53390 | Bajorek *et al.,* 2009 |
|  |  |  |  |  |
| pLVX TetOn-Advanced |  |  |  | Clontech/ Don Ayer gift |
| pLVX Tight-Puro |  |  |  | Clontech/  Don Ayer gift |
| pLVX mCherry | WISP20-61 | 180646 (Addgene) |  | Wenzel *et al.,* 2022 |
| pLVX CAPN7-mCherry | WISP20-50 | 180652 (Addgene) | Q9Y6W3 | Wenzel *et al.,* 2022 |
| pLVX CAPN7-V18D-mCherry | WISP22-44 | 193046 (Addgene) | Q9Y6W3 | This study |
| pLVX CAPN7-F98D-mCherry | WISP20-53 | 180653 (Addgene) | Q9Y6W3 | Wenzel *et al.,* 2022 |
| pLVX CAPN7-C290S-mCherry | WISP22-43 | 193047 (Addgene) | Q9Y6W3 | This study |
