# Supplementary material for: The Calpain-7 protease functions together with the ESCRT-III protein IST1 within the midbody to regulate the timing and completion of abscission
Source: eLife. 2023 Sep 29;12:e84515. doi: 10.7554/eLife.84515 (PMC10586806; doi:10.7554/eLife.84515)
Supplement: Supplementary file 3. [file elife-84515-supp3.docx]

**Supplementary File 3. siRNA Sequences**

| **siRNA** | **Protein Target** | **Sense Sequence** | **Source** | **Reference** |
| --- | --- | --- | --- | --- |
| siNT | Non-targeting | GCAAAUCUCCGAUCGUAGA | U of U Cores | Mackay *et al.*, 2010 |
| siCAPN7 | Calpain-7 | GCACCCAUACCUUUACAUU | U of U Cores | Wenzel *et al.,* 2022 |
| siNUP153 | Nup153 | GGACUUGUUAGAUCUAGUU | U of U Cores | Mackay *et al.,*, 2010 |
| siKATNA1 | Katanin p60 | GGACAGCACUCCCUUGAAA | U of U Cores | Wenzel *et al.,* 2022 |
| siSPAST | Spastin | GAACAGUGUGAAAGAGCUA | U of U Cores | Wenzel *et al.,* 2022 |
