# Supplementary material for: The Calpain-7 protease functions together with the ESCRT-III protein IST1 within the midbody to regulate the timing and completion of abscission
Source: eLife. 2023 Sep 29;12:e84515. doi: 10.7554/eLife.84515 (PMC10586806; doi:10.7554/eLife.84515)
Supplement: Supplementary file 4. [file elife-84515-supp4.docx]

**Supplementary File 4. Antibodies**

| **Target** | **Host Organism** | **Source** | **Product Number** | **Application^1^** | **Dilution Factor** |
| --- | --- | --- | --- | --- | --- |
| CAPN7 | Rabbit | Proteintech | 26985-1-AP | IF  WB | 1:500  1:4,000 |
| GAPDH | Mouse | Millipore | MAB374 | WB | 1:20,000 |
| IST1 | Rabbit | Sundquist Lab/ Covance | UT560 | IF | 1:1,000 |
| mCherry | Rabbit | Abcam | ab167453 | IF | 1:1,000 |
| RFP (mCherry) | Rat | ChromoTek | 5F8 | IF | 1:500 |
| RFP (mCherry) | Mouse | ChromoTek | 6G6 | WB | 1:1,000 |
| NUP153 (SA1) | Mouse | Brian Burke, Singapore | N/A | WB | 1:50 |
| NUP50 | Rabbit | Mackay, et al., 2010 | N/A | WB | 1:2,500 |
| α-TUBULIN (DM1A) | Mouse | Cell Signaling Technology | 3873S | IF | 1:2,000 |
| α-TUBULIN | Chicken | Synaptic Systems | 302 206 | IF | 1:1,000 |
| Myc | Mouse | EMD Millipore | Clone 4A6 | WB | 1:4,000 |
| Flag | Mouse | Sigma | M2 | WB | 1:5,000 |
| IRDye 800 CW,  Mouse | Donkey | Licor | 926-32212 | WB | 1:10,000 |
| IRDye 680,  Mouse | Donkey | Licor | 926-68072 | WB | 1:10,000 |
| IRDye 800 CW,  Rabbit | Donkey | LiCor | 926-32213 | WB | 1:10,000 |
| IRDye 680, Rabbit | Donkey | LiCor | 926-68073 | WB | 1:10,000 |
| DyLight 405 AffiniPure, Chicken IgG | Donkey | Jackson Immunoresearch | 703-475-155 | IF | 1:500 |
| Alexa Fluor 594 AffiniPure, Rat IgG | Donkey | Jackson Immunoresearch | 712-585-150 | IF | 1:500 |
| Alexa Fluor Plus 488, Rabbit IgG | Donkey | Thermo Fisher | A-32790 | IF | 1:1,000 |
| Alexa Fluor Plus 488, Mouse IgG | Donkey | Thermo Fisher | A-32766 | IF | 1:1,000 |
| Alexa Fluor Plus 594, Rabbit IgG | Donkey | Thermo Fisher | A-32754 | IF | 1:1,000 |

^1^IF = Immunofluorescence; WB = Western Blot
